# The global burden of tuberculosis attributable to diet high in processed meat from 1990 to 2021: findings from the Global Burden of Disease Study 2021

**DOI:** 10.3389/fnut.2025.1666550

**Published:** 2026-01-20

**Authors:** Linlin Wang, Honglu Wang, Yifei Wang, Chao Ma, Xinying Shi

**Affiliations:** 1 Department of Health Care Endocrinology and Metabolic Diseases, Central Hospital Affiliated with Shandong First Medical University, Jinan, Shandong, China; 2 Department of Cardiac Surgery, Central Hospital Affiliated with Shandong First Medical University, Jinan, Shandong, China; 3 Department of Pulmonary and Critical Care Medicine, Central Hospital Affiliated with Shandong First Medical University, Jinan, Shandong, China

**Keywords:** DALYs, diet high in processed meat, GBD 2021, health inequality, SDI, tuberculosis

## Abstract

**Background:**

Processed meats can increase the risk of tuberculosis (TB) infection by inducing chronic inflammation, disrupting the balance of the gut microbiota, and impairing metabolic health through their content of saturated fats, salt, and various additives. This study aims to analyze the global trends in the burden of TB attributable to diets high in processed meat (DHPM) from 1990 to 2021, and to explore the impacts of sex, age, and sociodemographic index (SDI) on these trends.

**Methods:**

Data were obtained from the global burden of disease study 2021 (GBD 2021). The DisMod-MR 2.1 model was used to integrate global data, focusing on two key indicators: disability-adjusted life years (DALYs) and mortality.

**Results:**

In 2021, the global number of DALYs due to TB attributable to DHPM was 78,891.94, and the number of deaths was 2,361.79, representing decreases of 4 and 8%, respectively, compared to 1990. The highest DALYs and death cases were observed in low-middle SDI regions, while the fastest increases occurred in low SDI regions. The age group with the highest DALYs cases for both males and females was 50–54 years, while the highest number of deaths occurred in the 55–59 age group, with males bearing a significantly higher burden than females. Approximately 47% of countries experienced an upward trend in DALYs and 43% in death cases. Countries with lower SDI levels bear a heavier TB burden, and the inequality has not shown significant improvement.

**Conclusion:**

Although the global burden of TB attributable to DHPM has shown an overall declining trend, marked regional disparities persist, with low SDI regions and males facing higher risks.

## Introduction

Tuberculosis (TB), caused by *Mycobacterium tuberculosis*, remains one of the leading infectious causes of morbidity and mortality worldwide, particularly affecting populations in low-income settings and individuals with impaired immunity (1–5). Despite global progress in TB control, substantial disparities persist, underscoring the need to identify modifiable risk factors that influence susceptibility and disease progression.

Dietary patterns have emerged as an important determinant of immune competence and infectious disease risk. Among dietary exposures, processed meat intake is of particular concern due to its high content of saturated fats, salt, nitrites, heme iron, and various chemical additives (6). A growing body of evidence indicates that these components can induce chronic low-grade inflammation, promote oxidative stress, and disrupt gut microbiota homeostasis—mechanisms that may compromise host defense against intracellular pathogens (7). Emerging mechanistic studies suggest that processed meat may suppress Th1-mediated immunity, impair macrophage bactericidal activity, and alter gut-lung immune crosstalk, all of which are essential for controlling *M. tuberculosis* infection (8). However, although processed meat consumption has been extensively linked to cardio-metabolic diseases and neoplasms, direct epidemiological evidence connecting processed meat exposure with TB burden remains scarce and fragmented.

Given this knowledge gap, a comprehensive global assessment is urgently needed to clarify whether high processed meat intake contributes to TB burden across regions with different socioeconomic conditions and demographic profiles. Existing country-level or small regional studies are insufficient for capturing global patterns or for comparing trends between populations.

To address the existing evidence gap, this study utilizes data from the global burden of disease (GBD) 2021 project. The dataset integrates comprehensive vital registration records, population surveys, and multi-source modeling outputs, and employs the DisMod-MR 2.1 system to generate standardized and comparable estimates for 204 countries and territories and 811 subnational locations from 1990 to 2021 (9, 10). These features enable robust estimation of the burden of TB attributable to diet high in processed meat (DHPM). The use of GBD 2021 is further justified by its most up-to-date and comprehensive global estimates, harmonized definitions that ensure valid cross-country comparisons, the ability to quantify population-attributable burden for assessing demographic and epidemiologic drivers, and the availability of SDI-stratified data essential for examining global inequalities.

Building on these strengths, this study systematically describes temporal trends in the burden of TB attributable to DHPM at global, regional, and national levels from 1990 to 2021, compares variations across sex, age, and sociodemographic index (SDI), and evaluates inequality patterns and future projections. By integrating dietary risk assessment with TB epidemiology, this work fills a critical evidence gap and provides an updated scientific foundation for developing nutrition-informed TB prevention strategies and designing targeted global health interventions.

## Methods

This study is based on comprehensive data from the GBD 2021 and systematically analyzes the burden of TB attributable to DHPM across 204 countries and territories, as well as 811 subnational locations, from 1990 to 2021 (9–12). Using the standardized GBD 2021 data integration approach, the analysis employed the DisMod-MR 2.1 model to synthesize data from 22,603 vital registration sources and 1,718 verbal autopsy datasets (9). A Bayesian meta-regression framework was applied to address data gaps and ensure internal consistency of TB estimates. The study focused on two core metrics—disability-adjusted life years (DALYs) and mortality. DALYs were calculated as the sum of years of life lost (YLL) due to premature death and years lived with disability (YLD), incorporating updated disability weights (DWs) to reflect the severity variations across different forms of TB (9).

In the GBD 2021 database, DHPM is defined as any consumption of processed meat that exceeds the theoretical minimum risk exposure level (TMREL), which is 0 g per day (13, 14). This means that any intake of processed meat is considered a risk exposure to DHPM. TB, caused by *M. tuberculosis*, includes both pulmonary and extra-pulmonary manifestations. The International Classification of Diseases 10th revision (ICD-10) codes are A10–A19.9, B90–B90.9, K67.3, K93.0, M49.0, and P37.0 (15).

To assess the drivers of TB burden changes, the Das Gupta decomposition method was used to quantitatively evaluate the relative contributions of population growth, aging, and epidemiological changes (11, 16). Furthermore, cross-national health inequalities were assessed using the slope index of inequality (SII) and the concentration index (CI) (17).

For future burden projections, the Nordpred model was applied, incorporating age-period-cohort (APC) effects to extrapolate trends, and external validation was conducted to verify model performance (12). It is important to note that the Nordpred model assumes that the trends observed over the past decades will continue into the future (18). However, this assumption may not be entirely accurate, as new public health interventions, socioeconomic changes, or advances in medical technology may emerge and alter future TB trends. Changes in global health policies, economic crises, climate change, and other factors may also influence the trajectory of tuberculosis, yet these factors are difficult to fully incorporate into the model. Moreover, in the coming decades, significant shifts in global population structure and levels of economic development may occur, particularly in low SDI regions. Such changes may alter the distribution of the TB burden, thereby increasing the uncertainty of future predictions (18).

## Results

### Global and regional burden of TB attributable to DHPM

**Figure 1:**
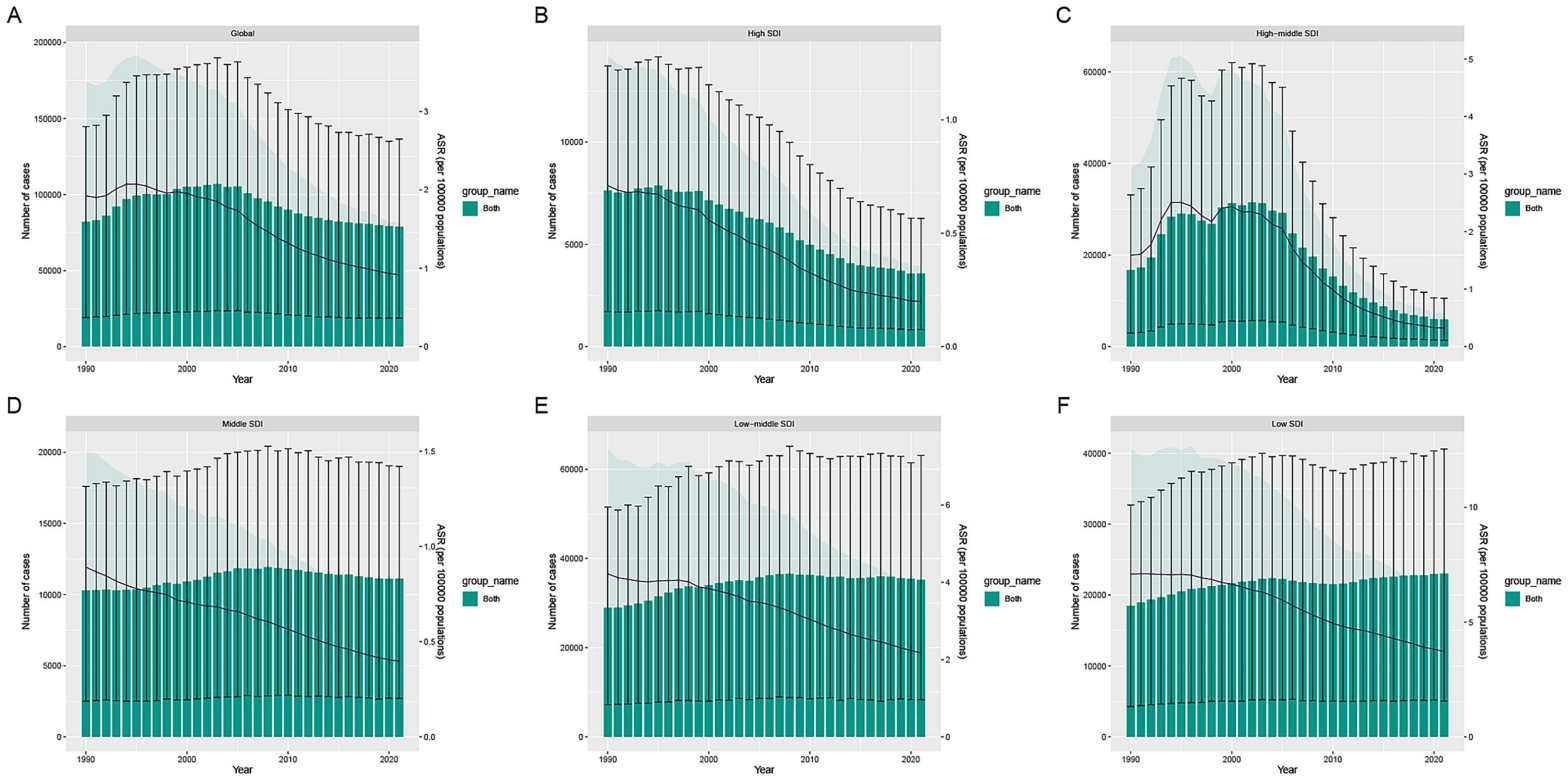
**(A)** Global DALYs cases and ASDR of tuberculosis attributable to DHPM from 1990 to 2021. **(B-F)** DALYs cases and ASDR of tuberculosis attributable to DHPM across the 5 SDI regions from 1990 to 2021.

In 2021, the global burden of TB attributable to DHPM remained considerable, although modest declines were observed compared with 1990. Worldwide, DALYs decreased by 4% and deaths by 8%, reaching 78,891.94 DALYs and 2,361.79 deaths. Low-middle SDI regions continued to experience the largest absolute burden, contributing nearly half of all global DALYs. In contrast, the fastest increases were observed in low SDI regions, where DALYs and deaths rose by 24% and 18%, respectively. Notably, southern sub-Saharan Africa exhibited the steepest regional escalation, with DALYs and deaths increasing by 138 and 146%, respectively (Figure 1A-F).

Globally, the age-standardized DALYs rate (ASDR) was 0.92 per 100,000 in 2021 (EAPC: −2.88), and the age-standardized mortality rate (ASMR) was 0.02751 per 100,000 (EAPC: −3.14), demonstrating consistent long-term declines. Despite these improvements, low SDI regions still showed the highest ASDR and ASMR, underscoring persistent socioeconomic inequalities (Supplementary Figure S1 and Tables 1, 2).

**Table 1:** Disability-adjusted life years (DALYs) and age-standardized DALY rate (ASDR) of TB attributable to DHPM in 1990 and 2021, and the PC and EAPC from 1990 to 2021.

Location	1990_DALYs cases (95% UI)	2021_DALYs cases (95% UI)	Percentage change	1990_ASDR_per 100,000 (95% UI)	2021_ASDR_per 100,000 (95% UI)	EAPC (95% CI)
Andean Latin America	210.98 (47.93–381.1)	135.72 (30.91–236.4)	−0.36	0.91 (0.21–1.62)	0.22 (0.05–0.38)	−4.55 (−4.85–−4.25)
Australasia	41.32 (9.19–72.78)	40.25 (9.27–72.56)	−0.03	0.18 (0.04–0.31)	0.08 (0.02–0.15)	−2.49 (−2.81–−2.17)
Caribbean	101.98 (23.98–251.7)	119.83 (27.01–286.23)	0.18	0.36 (0.09–0.9)	0.23 (0.05–0.55)	−0.98 (−1.33–−0.63)
Central Asia	644.09 (135.11–1157.96)	818.64 (185.78–1458.72)	0.27	1.21 (0.26–2.17)	0.83 (0.19–1.48)	−2.29 (−3.17–−1.39)
Central Europe	1107.26 (263.9–1929.58)	783.1 (188.62–1427.11)	−0.29	0.75 (0.18–1.31)	0.44 (0.11–0.81)	−1.69 (−1.99–−1.39)
Central Latin America	1108.36 (264.08–1923.74)	692.44 (164.37–1241.84)	−0.38	1.11 (0.26–1.9)	0.26 (0.06–0.47)	−4.72 (−5.19–−4.25)
Central Sub-Saharan Africa	5198.46 (1164.48–10231.72)	7393.74 (1532.18–14065.4)	0.42	18.41 (4.13–35.6)	9.73 (2.03–18.29)	−2.17 (−2.3–−2.04)
East Asia	5411.19 (1325.53–9582.14)	3804.04 (972.88–6697.09)	−0.3	0.58 (0.14–1.01)	0.18 (0.05–0.31)	−3.6 (−3.72–−3.48)
Eastern Europe	12746.74 (2073.17–26581.61)	2945.92 (635.58–5441.83)	−0.77	4.7 (0.75–9.85)	1.01 (0.22–1.85)	−6.63 (−8.25–−4.99)
Eastern Sub-Saharan Africa	4732.52 (1043.27–8427.12)	5351.32 (1257.7–9661.7)	0.13	5.55 (1.25–9.8)	2.68 (0.64–4.78)	−2.6 (−2.71–−2.48)
Global	82125.16 (19091.73–144740.89)	78891.94 (18798.65–136564.61)	−0.04	1.92 (0.45–3.38)	0.92 (0.22–1.58)	−2.88 (−3.21–−2.55)
High-income Asia Pacific	3163.63 (754.31–5477.94)	1180.46 (295.9–2027.39)	−0.63	1.54 (0.37–2.66)	0.26 (0.06–0.44)	−6.27 (−6.65–−5.88)
High-income North America	1615.04 (359.01–2934.71)	1018.15 (241.02–1829.92)	−0.37	0.49 (0.11–0.9)	0.2 (0.04–0.35)	−3.51 (−3.86–−3.16)
High-middle SDI	16727.36 (2967.07–33160.74)	5938.61 (1390.07–10579.16)	−0.64	1.59 (0.28–3.13)	0.32 (0.07–0.58)	−6.62 (−7.81–−5.42)
High SDI	7631.23 (1706.87–13714.59)	3570.9 (826.49–6267.37)	−0.53	0.71 (0.16–1.28)	0.2 (0.05–0.36)	−4.55 (−4.8–−4.3)
Low-middle SDI	28938.84 (7237.52–51534.6)	35209.19 (8339.66–63118.6)	0.22	4.22 (1.07–7.46)	2.17 (0.52–3.89)	−2.18 (−2.36–−2)
Low SDI	18490.16 (4286.52–32695.97)	23017.68 (5065.88–40592.63)	0.24	7.09 (1.66–12.56)	3.72 (0.83–6.54)	−2.29 (−2.46–−2.12)
Middle SDI	10300.35 (2512.45–17609.64)	11122.02 (2709.94–19017.6)	0.08	0.89 (0.22–1.5)	0.4 (0.1–0.68)	−2.54 (−2.66–−2.42)
North Africa and Middle East	1152.49 (259.1–2218.01)	1447.2 (349.13–2576.02)	0.26	0.61 (0.14–1.2)	0.26 (0.06–0.47)	−2.82 (−2.88–−2.75)
Oceania	83.92 (19.59–156.02)	127.4 (27.83–225.16)	0.52	2.25 (0.53–4.15)	1.33 (0.29–2.36)	−1.72 (−1.76–−1.68)
South Asia	31516.48 (7806.81–56451.54)	35338.45 (8222–63535.65)	0.12	4.82 (1.2–8.51)	2.14 (0.5–3.84)	−2.74 (−2.93–−2.55)
Southeast Asia	3024.93 (704.09–5226.89)	3921.94 (917.88–6656.47)	0.3	1.1 (0.26–1.9)	0.56 (0.13–0.95)	−2.17 (−2.23–−2.12)
Southern Latin America	432.91 (93.91–773.93)	401.49 (95.3–724.71)	−0.07	0.93 (0.2–1.66)	0.49 (0.12–0.89)	−1.75 (−1.94–−1.57)
Southern Sub-Saharan Africa	787.25 (172.51–1407.96)	1872.53 (455.26–3247.96)	1.38	2.39 (0.54–4.25)	2.71 (0.66–4.71)	0.97 (0.12–1.84)
Tropical Latin America	486.49 (118.59–852.2)	874.23 (201.09–1532.18)	0.8	0.44 (0.11–0.78)	0.33 (0.08–0.58)	−0.82 (−1–−0.63)
Western Europe	2522.28 (551.05–4592.18)	848.76 (185.88–1502.48)	−0.66	0.46 (0.1–0.85)	0.1 (0.02–0.19)	−4.9 (−5–−4.79)
Western sub-Saharan Africa	6036.83 (1387.41–10859.7)	9776.33 (2335.06–17150.96)	0.62	6.24 (1.45–11.08)	4.19 (1.03–7.35)	−1.35 (−1.57–−1.13)

DHPM, diet high in processed meat; EAPC, estimated annual percentage change; PC, percentage change; TB, tuberculosis.

**Table 2:** Deaths and age-standardized mortality rate (ASMR) of TB attributable to DHPM in 1990 and 2021, and the PC and EAPC from 1990 to 2021.

Location	1990_Death cases (95% UI)	2021_Death cases (95% UI)	Percentage change	1990_ASMR_per 100,000 (95% UI)	2021_ASMR_per 100,000 (95% UI)	EAPC (95% CI)
Andean Latin America	6.57 (1.58–11.51)	4.21 (0.97–7.35)	−0.36	0.03092 (0.00761–0.05359)	0.00699 (0.00162–0.01226)	−4.75 (−5.06–−4.45)
Australasia	1.76 (0.4–3.11)	1.75 (0.4–3.13)	−0.01	0.00748 (0.0017–0.01323)	0.00311 (0.00072–0.00555)	−2.85 (−3.17–−2.53)
Caribbean	3 (0.7–7.87)	3.33 (0.77–8.14)	0.11	0.01126 (0.00266–0.02953)	0.00631 (0.00145–0.01548)	−1.38 (−1.72–−1.05)
Central Asia	16.46 (3.56–29.16)	20.02 (4.61–35.32)	0.22	0.03226 (0.00706–0.05706)	0.02115 (0.00486–0.03733)	−2.36 (−3.2–−1.52)
Central Europe	36.63 (8.89–63.25)	24.33 (5.85–43.08)	−0.34	0.0249 (0.00599–0.04316)	0.01252 (0.00296–0.02239)	−2.21 (−2.49–−1.94)
Central Latin America	32.15 (7.6–55.04)	18.57 (4.44–32.73)	−0.42	0.0361 (0.0085–0.06089)	0.00719 (0.00172–0.01262)	−5.31 (−5.77–−4.84)
Central Sub-Saharan Africa	143.67 (32.19–277.73)	191.42 (39.89–364.2)	0.33	0.60051 (0.13253–1.14419)	0.3051 (0.0646–0.57204)	−2.32 (−2.47–−2.18)
East Asia	177.96 (44.52–307.68)	108.68 (26.67–197.49)	−0.39	0.02206 (0.0056–0.03802)	0.00507 (0.00125–0.00913)	−4.63 (−4.79–−4.47)
Eastern Europe	327.41 (56.8–659.14)	79.83 (17.33–146.48)	−0.76	0.11786 (0.0201–0.23841)	0.02572 (0.0056–0.04725)	−6.5 (−8.05–−4.92)
Eastern Sub-Saharan Africa	148.59 (33.66–256.75)	166.89 (40.04–296.08)	0.12	0.20773 (0.0484–0.35922)	0.10285 (0.02511–0.18315)	−2.51 (−2.62–−2.39)
Global	2561.69 (612.48–4474.85)	2361.79 (570.68–4103.82)	−0.08	0.06393 (0.01539–0.11137)	0.02751 (0.00663–0.04774)	−3.14 (−3.44–−2.84)
High-income Asia Pacific	123.78 (30.45–213.22)	67.9 (17.75–115.66)	−0.45	0.06165 (0.01518–0.10617)	0.01195 (0.00303–0.02042)	−5.81 (−6.2–−5.42)
High-income North America	59.89 (14.1–109.99)	33.03 (7.98–58.4)	−0.45	0.01721 (0.00404–0.03167)	0.00564 (0.00135–0.01006)	−4.26 (−4.66–−3.85)
High-middle SDI	465.26 (87.19–895.58)	171.97 (40.66–309.08)	−0.63	0.0454 (0.00857–0.08678)	0.00903 (0.00213–0.01628)	−6.54 (−7.62–−5.45)
High SDI	296.88 (67.74–520.22)	153 (37.41–262.14)	−0.48	0.02681 (0.00608–0.04697)	0.00716 (0.00172–0.01236)	−4.74 (−5–−4.48)
Low-middle SDI	914.9 (229.72–1620.43)	1043.76 (252.68–1857.95)	0.14	0.15315 (0.03791–0.26917)	0.07094 (0.01722–0.12575)	−2.57 (−2.74−−2.4)
Low SDI	561.42 (132.55–991.07)	663.78 (149.67–1169.02)	0.18	0.25038 (0.06056–0.44049)	0.12757 (0.02973–0.22332)	–−2.4 (−2.58–−2.23)
Middle SDI	322.03 (80.3–547.48)	328.25 (79.23–566.12)	0.02	0.03227 (0.00814–0.05463)	0.01223 (0.00294–0.02099)	−3.07 (−3.2–−2.95)
North Africa and Middle East	37.46 (8.55–75.19)	40.39 (9.89–74.04)	0.08	0.02304 (0.00531–0.04795)	0.00831 (0.00205–0.01535)	−3.34 (−3.38–−3.29)
Oceania	2.27 (0.54–4.22)	3.36 (0.73–5.91)	0.48	0.0742 (0.01799–0.13492)	0.0429 (0.00945–0.07559)	−1.78 (−1.82–−1.74)
South Asia	986.16 (245.64–1743.55)	1038.6 (247.22–1834.21)	0.05	0.17565 (0.04353–0.30826)	0.06892 (0.01651–0.12135)	−3.19 (−3.39–−3)
Southeast Asia	102.13 (24.44–176.87)	126.96 (30.47–212.35)	0.24	0.04359 (0.01072–0.07524)	0.0201 (0.00488–0.03368)	−2.44 (−2.53–−2.34)
Southern Latin America	14.47 (3.29–25.95)	13.65 (3.28–24.76)	−0.06	0.03144 (0.00717–0.05626)	0.01605 (0.00385–0.0291)	−1.93 (−2.09–−1.77)
Southern Sub-Saharan Africa	21.47 (4.85–38.38)	52.9 (13.02–91.89)	1.46	0.07324 (0.01681–0.13031)	0.08514 (0.02124–0.14612)	0.99 (0.19–1.8)
Tropical Latin America	13.36 (3.27–23.12)	24.02 (5.65–41.88)	0.8	0.01352 (0.00332–0.02323)	0.00916 (0.00216–0.01598)	−1.16 (−1.31–−1.01)
Western Europe	109.55 (24.19–192.89)	42.33 (9.74–75)	−0.61	0.01886 (0.00414–0.03339)	0.00411 (0.00092–0.00724)	−5.12 (−5.26–−4.99)
Western Sub-Saharan Africa	196.95 (45.7–348.6)	299.62 (73.92–529.36)	0.52	0.23445 (0.05556–0.41378)	0.15661 (0.0392–0.27574)	−1.39 (−1.6–−1.18)

DHPM, diet high in processed meat; EAPC, estimated annual percentage change; PC, percentage change; TB, tuberculosis‌.

### National burden of TB attributable to DHPM

**Figure 2:**
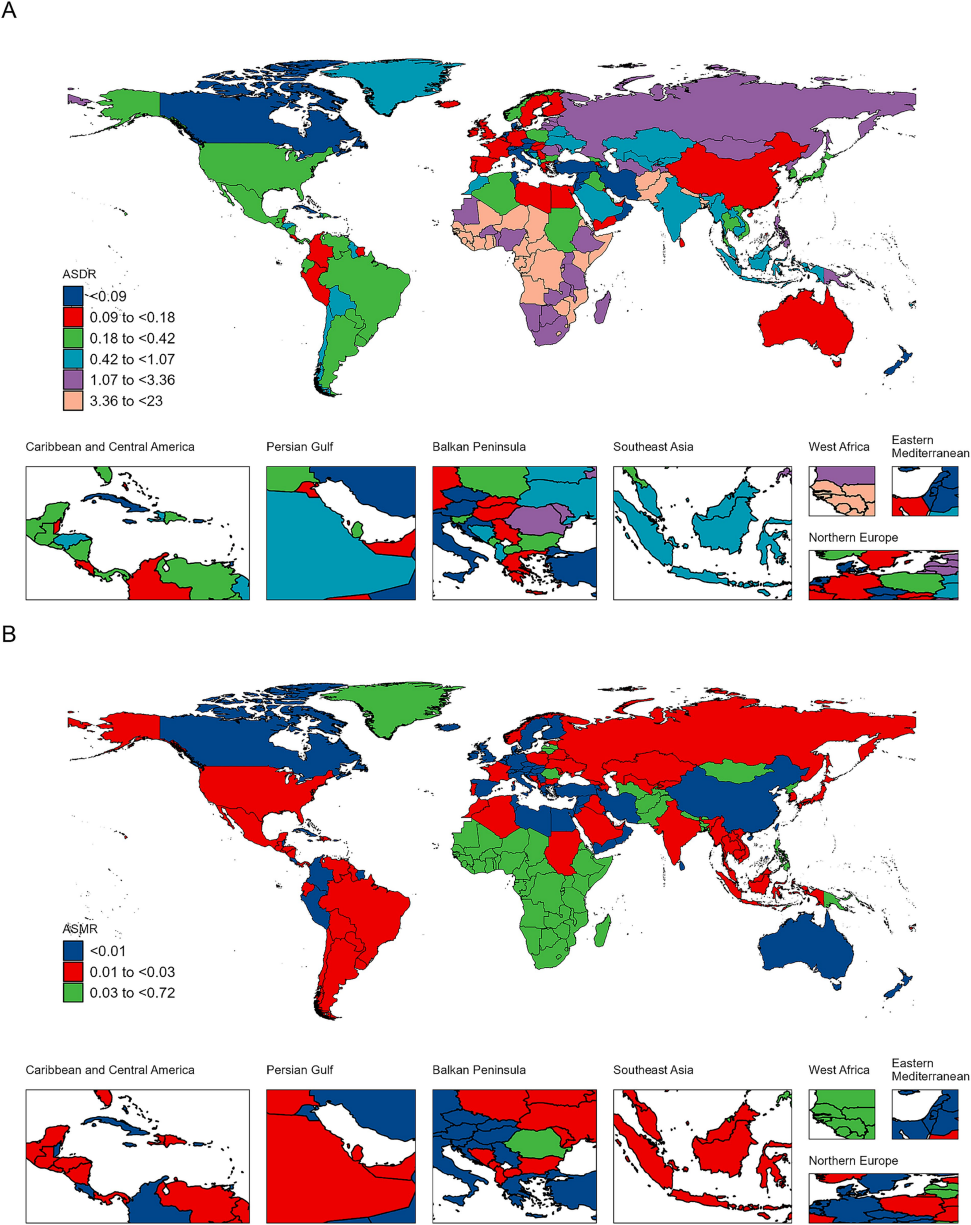
ASDR **(A)** and ASMR **(B)** of TB attributable to DHPM per 100,000 population in 2021, by country. ASDR, age-standardized DALYs rate; ASMR, age-standardized mortality rate; DHPM, diet high in processed meat; TB, tuberculosis.

National patterns revealed substantial heterogeneity. Nearly 47% of countries showed increasing DALYs and 43% showed increasing deaths between 1990 and 2021. The most rapid national-level increases occurred in Lesotho, Djibouti, and Ghana, each experiencing more than a two-fold rise (Figure 2A).

Similarly, several countries demonstrated rising age-standardized rates. Lesotho and Zimbabwe showed the fastest growth in both ASDR and ASMR, with positive EAPCs exceeding 2% per year, while Ukraine also exhibited upward trends. These findings highlight countries where TB control efforts face continuing challenges despite global progress (Figure 2B and Supplementary Tables S1, S2).

### Age and sex differences

**Figure 3:**
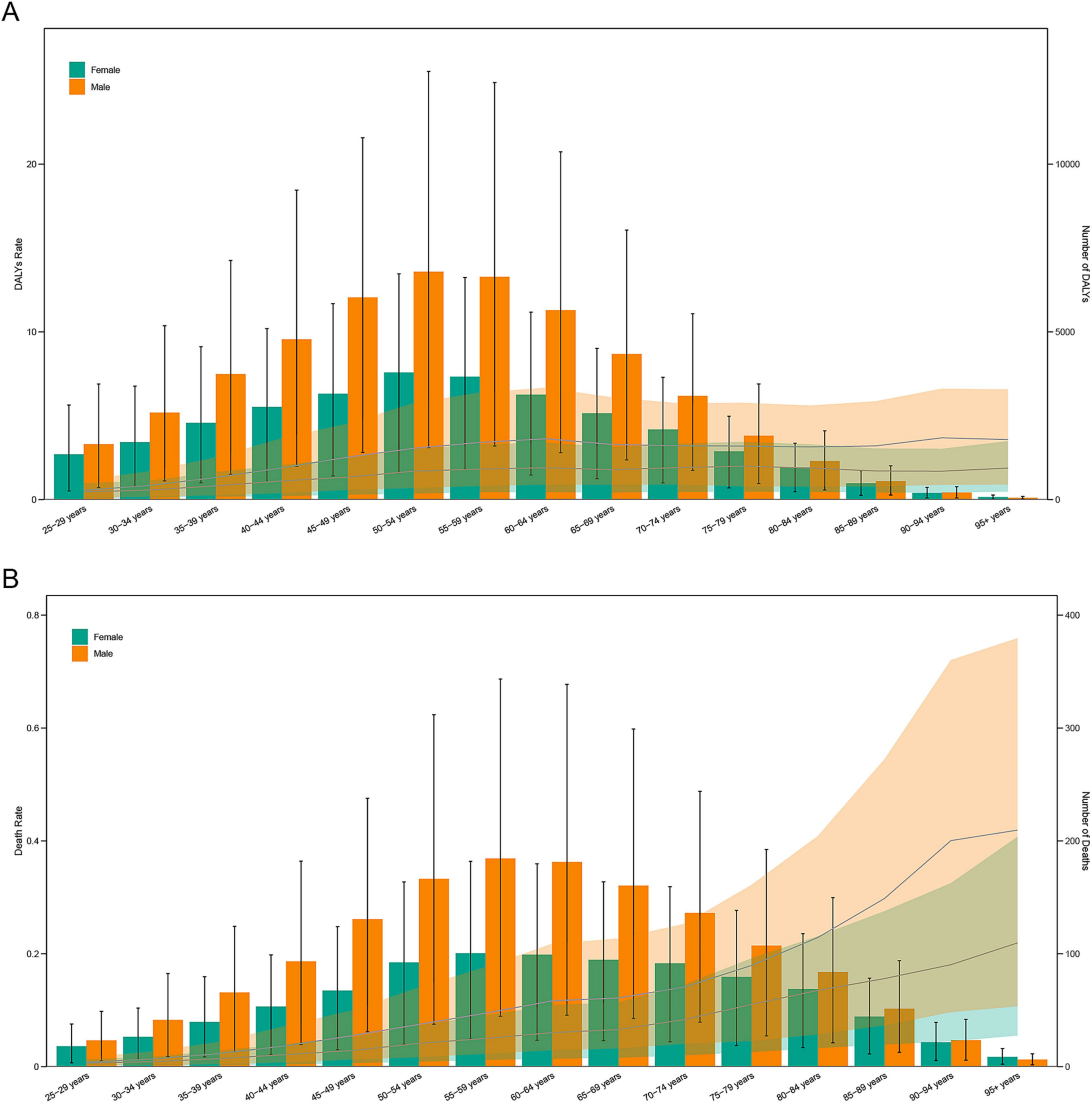
Age-specific numbers and rates of DALYs **(A)** and deaths **(B)** of TB attributable to DHPM by age and sex in 2021. DALYs, disability-adjusted life years; DHPM, diet high in processed meat; TB, tuberculosis.

Age- and sex-specific analyzes revealed distinct patterns. In 2021, DALYs peaked at ages 50–54 years for both sexes, suggesting a substantial burden during economically active midlife years. Mortality peaked slightly later, at 55–59 years, with death rates rising steadily with age (Figure 3A).

Across all age groups, males consistently exhibited higher DALYs and mortality rates than females, with the highest DALYs rate in males appearing in the 90–94 years group, and in females in the 75–79 years group. These differences may reflect variations in exposure, comorbidities, and behavioral risk factors (Figure 3B).

### Association between TB attributable to DHPM burden and SDI

**Figure 4:**
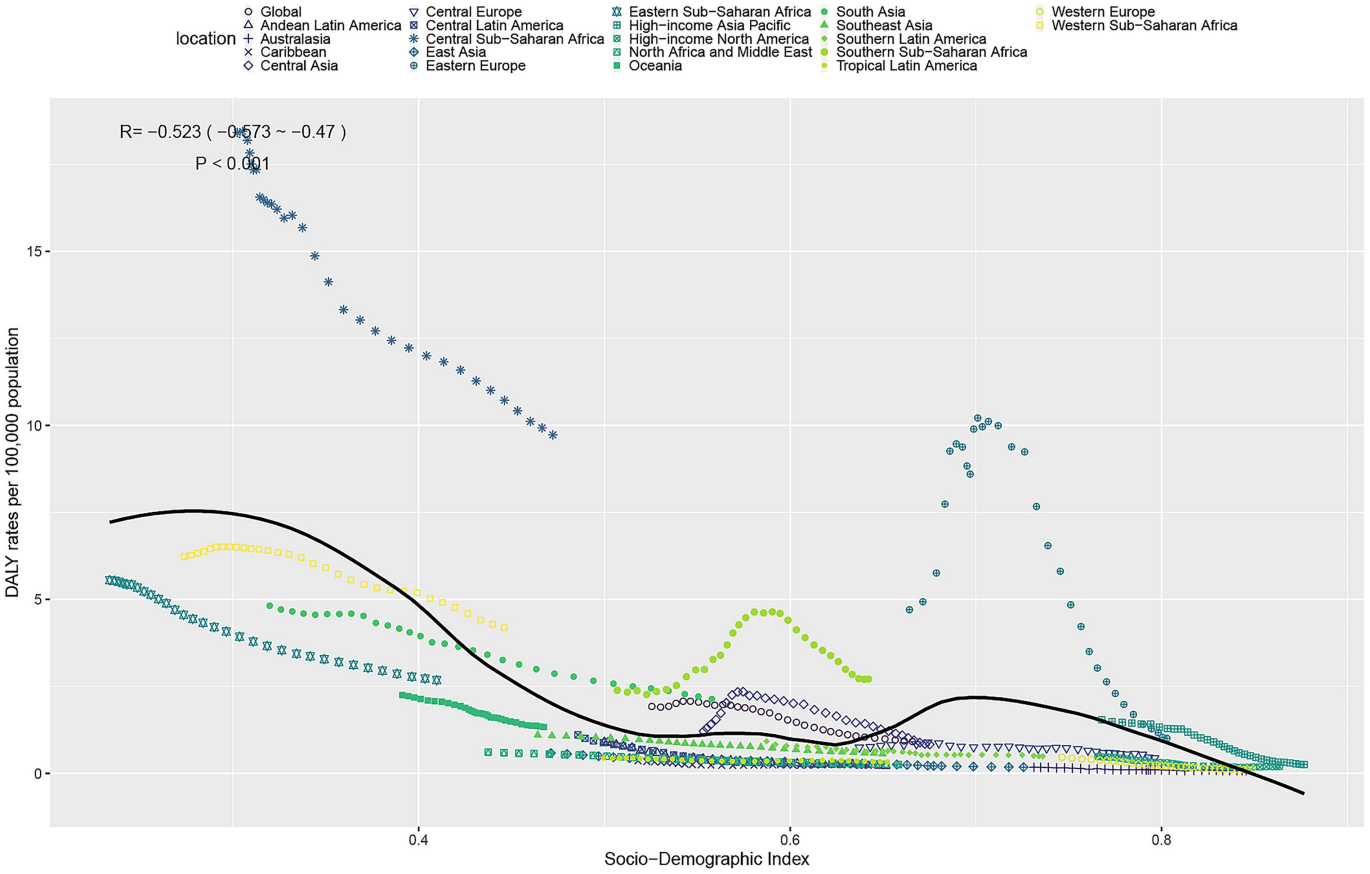
ASDR of TB attributable to DHPM in 21 GBD regions by SDI, 1990–2021. ASDR, age-standardized DALYs rate; DHPM, diet high in processed meat; TB, tuberculosis.

A strong negative correlation was observed between TB burden and SDI. As SDI increased, both ASDR and ASMR declined (Figure 4 and Supplementary Figure S2). However, several regions demonstrated higher-than-expected burden relative to SDI—including central and southern sub-Saharan Africa and eastern Europe—indicating that socioeconomic development alone does not fully explain disease patterns. Conversely, regions such as Oceania and eastern sub-Saharan Africa showed lower-than-expected rates, suggesting potential benefits from health interventions or changing dietary patterns.

### Decomposition of TB attributable to DHPM burden changes

**Figure 5:**
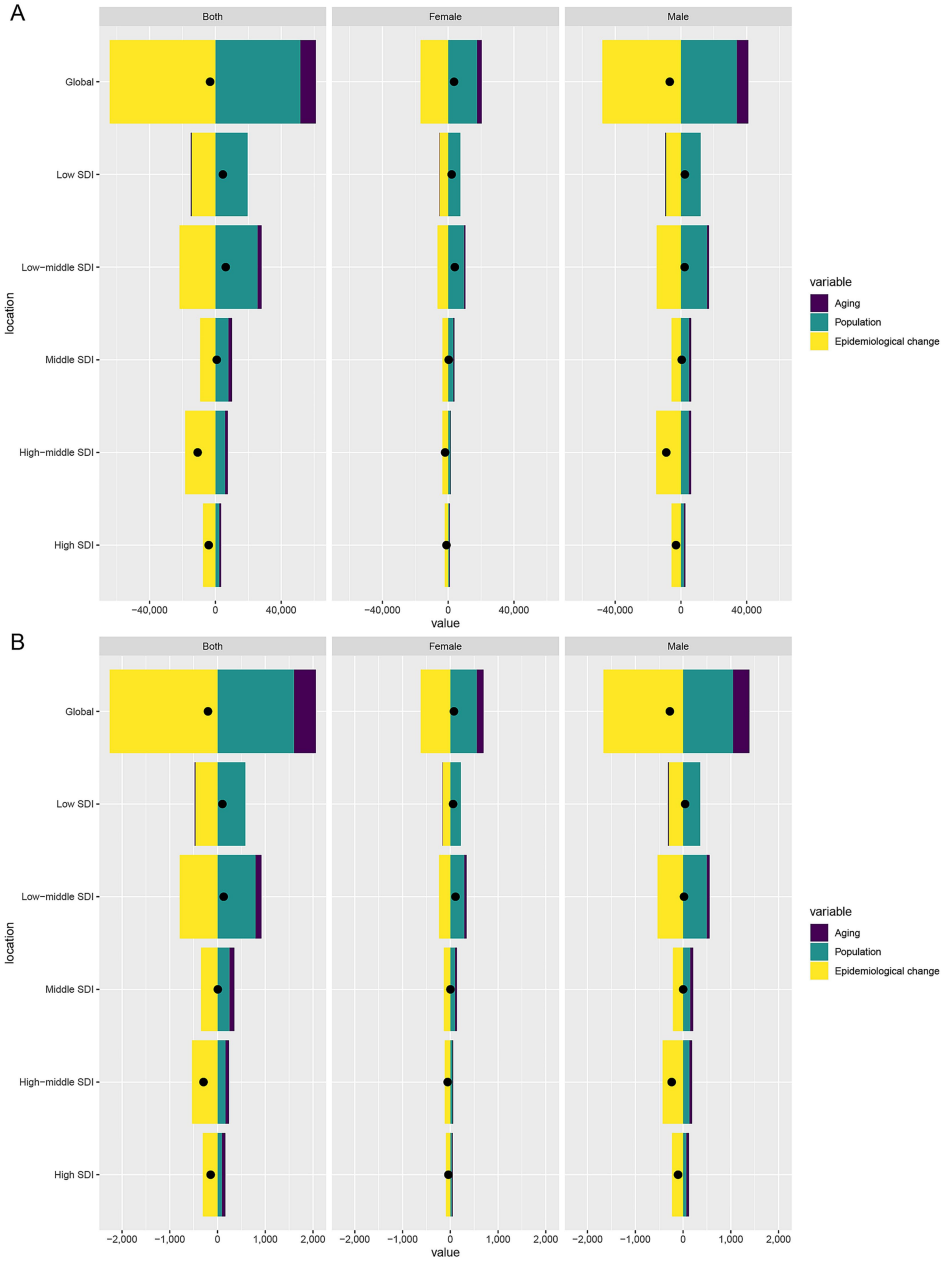
Decomposition analysis of changes in DALYs **(A)** and deaths **(B)** of TB attributable to DHPM between 1990 and 2021 across SDI regions. DALYs, disability-adjusted life years; DHPM, diet high in processed meat; TB, tuberculosis.

From 1990 to 2021, global DALYs attributable to DHPM showed a slight net reduction, but decomposition analysis revealed substantial underlying shifts. Population aging and growth strongly contributed to increased DALYs, whereas epidemiological improvements—including reduced exposure and better TB control—offset these increases and were the primary drivers of the net decline (Figure 5A).

The sex-specific decomposition indicated that DALYs among males decreased overall, while DALYs among females increased, driven largely by demographic expansion. A similar pattern was observed for mortality, with epidemiological changes mitigating increases but demographic pressures sustaining a substantial residual burden (Figure 5B).

### Cross-country inequality

**Figure 6:**
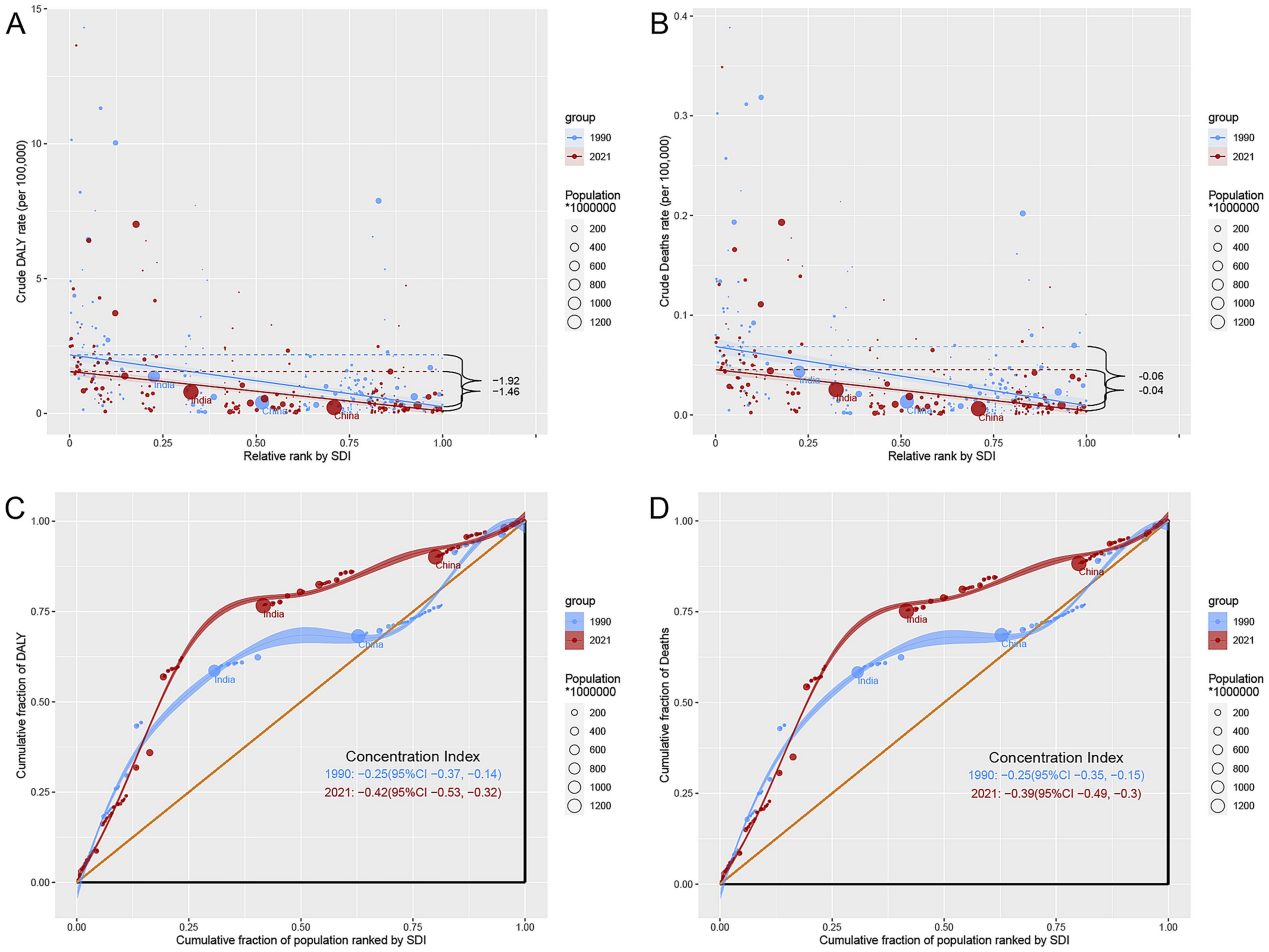
Health inequality analysis of DALYs and mortality in TB attributable to DHPM in 1990 and 2021 across the world. **(A)** Health inequality regression curves for DALYs. **(B)** Health inequality regression curves for mortality. **(C)** Concentration curves for DALYs. **(D)** Concentration curves for mortality. DALYs, disability-adjusted life years; DHPM, diet high in processed meat, TB, tuberculosis.

Inequality analyzes showed that countries with lower SDI continue to bear disproportionately higher burdens. Although the SII for DALYs and mortality changed only slightly from 1990 to 2021, the persistent negative CI demonstrates that TB burden remains concentrated in lower-income populations, with no meaningful narrowing of inequality over the past three decades (Figures 6A–D).

### Projections to 2045

**Figure 7:**
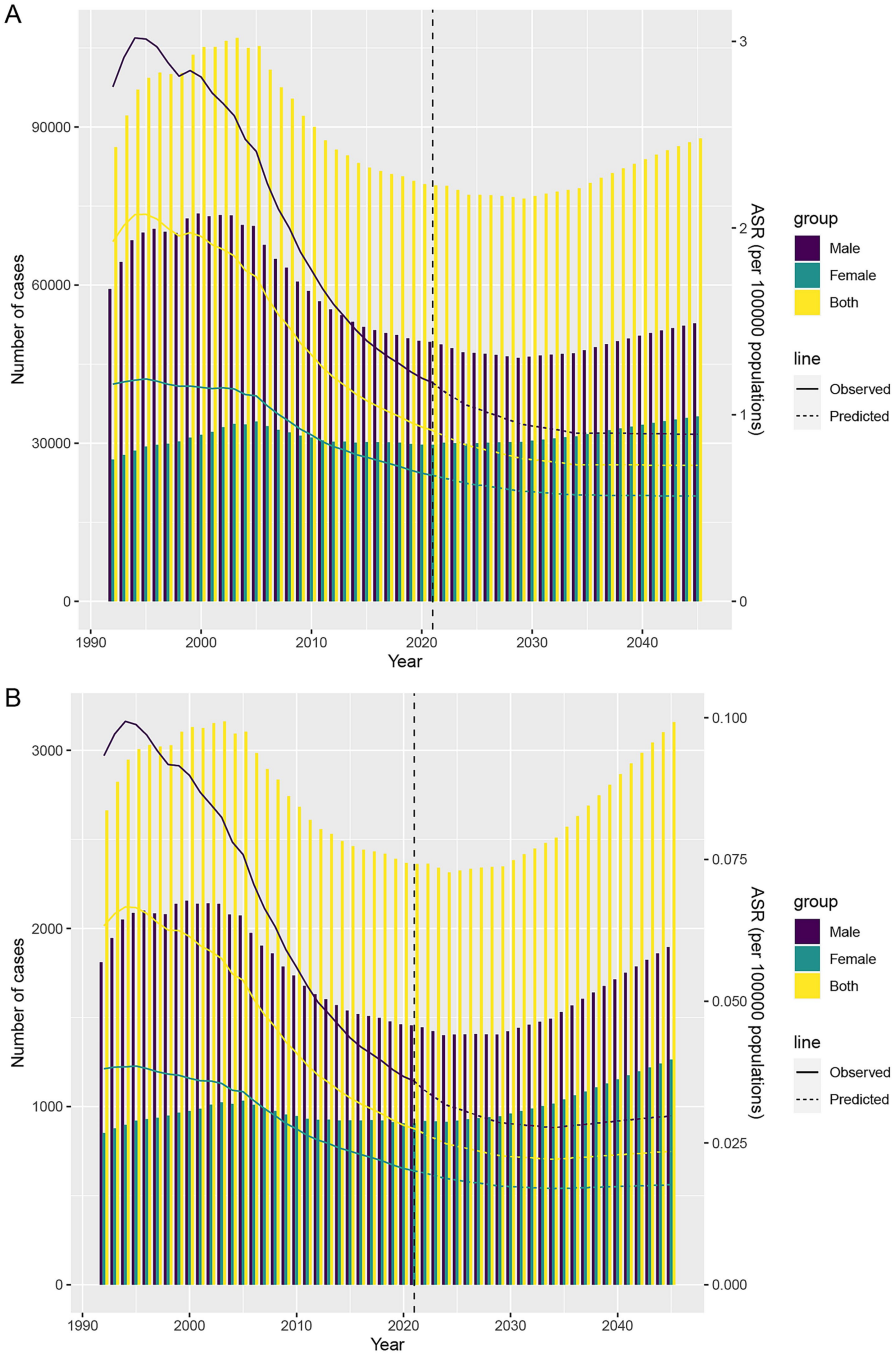
Projections of the temporal trends of the number of DALYs cases, death cases, ASDR, and ASMR of TB attributable to DHPM globally up to 2045. **(A)** The number and ASDR of TB attributable to DHPM by year and gender. **(B)** The number and ASMR of TB attributable to DHPM by year and gender. ASDR, age-standardized DALYs rate; ASMR, age-standardized mortality rate; DALYs, disability-adjusted life years; DHPM, diet high in processed meat; TB, tuberculosis.

Forecasts indicate that ASDR and ASMR will continue declining through 2045; however, absolute DALYs and deaths are projected to first decrease and then rise, driven largely by population aging. Males are projected to maintain a notably higher burden than females. By 2045, male DALYs are expected to reach 52,751, compared with 35,079 in females, highlighting the need for sex-specific intervention strategies (Figures 7A,B).

## Discussion

This study provides updated global evidence on the association between DHPM and the TB burden, extending previous literature by incorporating long-term trends and socioeconomic inequalities across 204 countries and territories. While several biological pathways suggest that processed meat intake may influence immune and metabolic functions relevant to TB susceptibility, it is important to emphasize that current evidence remains associative rather than causal. The immunological mechanisms—including nitrite-induced T-cell dysfunction, oxidative stress, gut microbiota dysbiosis, and chronic low-grade inflammation—represent plausible but not yet directly confirmed pathways linking DHPM to TB risk. Further experimental and longitudinal studies are needed to validate these mechanisms (19, 20).

Our findings demonstrate that although the global DALYs and deaths due to TB attributable to DHPM have declined since 1990, pronounced regional disparities persist. Low and low-middle SDI regions bear the heaviest burden and exhibit the fastest increases. These patterns are consistent with previous GBD-based studies showing that TB outcomes are strongly influenced by social determinants, nutritional adequacy, and health system performance rather than by dietary exposures alone (21–23). Compared with studies linking processed meat intake to other chronic diseases, our results suggest that DHPM-related TB burden is more sensitive to contextual factors such as poverty, food insecurity, household crowding, HIV prevalence, and limited access to high-quality TB diagnosis and treatment (23).

The cross-country differences observed in our analysis likely reflect the joint contribution of health system capacity, nutritional status, and comorbidities. Countries with weak primary healthcare networks and fragmented referral systems often have delayed TB diagnosis, poor treatment adherence, and insufficient drug supply chains, which may amplify the population-level impact of dietary and environmental risk factors (22, 23). In addition, undernutrition—particularly deficiencies in micronutrients such as vitamins A, D, and zinc—could interact with high processed meat intake and further impair immune function (24–26). Comorbid conditions prevalent in many low-SDI settings, such as HIV infection and diabetes, may also exacerbate vulnerability to TB, consistent with evidence showing additive or synergistic effects between metabolic dysregulation and impaired immunity (27–30).

Consistent with previous TB epidemiology research, we observed substantially higher DALYs and mortality in males across most age groups. This sex gap has also been documented in recent GBD and WHO reports, and may reflect differences in occupational exposure, healthcare-seeking behavior, lifestyle factors, and underlying comorbidities (31–33). Our results also align with literature showing that middle-aged and older adults bear the highest burden of TB-related disability and deaths.

Importantly, we avoid causal interpretations in the context of ecological GBD analyzes. While our findings highlight consistent associations between DHPM and TB burden across multiple regions and time periods, they should not be interpreted as evidence that processed meat “causes” TB. Rather, DHPM may serve as a marker of broader dietary patterns or socioeconomic conditions that influence TB risk and outcomes. Residual confounding due to smoking, alcohol intake, air pollution, and access to healthcare cannot be fully excluded.

From a public health perspective, our results reinforce the need for targeted TB prevention strategies aligned with the sustainable development goals (SDGs), particularly SDG 3.3. First, nutritional guidance that promotes reduced processed meat intake and improved dietary quality may support TB prevention, especially in vulnerable populations. Second, the persistent inequality between SDI groups indicates that strengthening health system performance—expanding diagnostic coverage, ensuring treatment completion, and improving social protection programs—is essential for reducing TB burden. Third, the projected slow decline in ASDR and ASMR suggests that current interventions may be insufficient to meet global TB elimination targets without intensified resource allocation and multi-sectoral collaboration.

Finally, several limitations should be acknowledged. First, the ecological nature of GBD estimates precludes individual-level causal inference. Second, the accuracy of DHPM exposure assessment is constrained by variability in dietary survey methods across countries. In addition, some regions may still have incomplete or uncertain estimates of TB burden.

## Conclusion

This study provides a comprehensive assessment of the global burden of TB attributable to DHPM over the past three decades. Although a gradual global decline was observed, substantial regional inequalities persist, with low SDI and low-middle SDI regions continuing to experience disproportionate disease burdens. The demographic distribution further highlights that middle-aged and older adults, particularly men, remain the most affected groups, and nearly half of all countries show a rising trend. These findings underscore the importance of addressing diet-related risks in TB prevention and control. Reducing processed meat intake—through strengthened nutrition education, food policy measures, and public awareness—may serve as a complementary strategy for mitigating TB risk, especially in high-burden settings. At the same time, persistent disparities across socioeconomic strata emphasize the need to reinforce targeted TB control strategies, improve healthcare access, and allocate resources to high-burden, low SDI regions. Looking ahead, although age-standardized burden is projected to decline slowly, absolute DALYs and deaths may rise again due to population aging and growth. Focused interventions in vulnerable regions and populations will be essential to accelerate progress toward global TB reduction goals.

## Data Availability

The original contributions presented in the study are included in the article/Supplementary material, further inquiries can be directed to the corresponding author/s.
