# Supplementary material for: The global burden of tuberculosis attributable to diet high in processed meat from 1990 to 2021: findings from the Global Burden of Disease Study 2021
Source: Front Nutr. 2026 Jan 20;12:1666550. doi: 10.3389/fnut.2025.1666550 (PMC12864051; doi:10.3389/fnut.2025.1666550)
Supplement: Supplementary file 1 [file Table_1.pdf]

Location	1990_DALYs cases (95% UI)	2021_DALYs cases (95% UI)	Percentage change	1990_ASDR_per 100000(95% UI)	2021_ASDR_per 100 000(95% UI)	EAPC (95% CI)
Afghanistan	336.01 (78.05-688.79)	498.63 (117.9-1007.21)	0.48	4.68 (1.1-9.59)	3.46 (0.83-6.97)	-0.94 (-1.05--0.82)
Albania	44.05 (8.28-94.85)	27.3 (5.52-58)	-0.38	1.98 (0.38-4.21)	0.7 (0.14-1.52)	-2.96 (-3.14--2.78)
Algeria	82.21 (17.67-150.99)	102.99 (23.92-200.3)	0.25	0.64 (0.13-1.16)	0.27 (0.06-0.52)	-2.75 (-2.9--2.59)
American Samoa	0.15 (0.04-0.29)	0.16 (0.04-0.29)	0.07	0.5 (0.12-0.92)	0.33 (0.08-0.58)	-1.77 (-1.97--1.56)
Andorra	0.05 (0.01-0.1)	0.04 (0.01-0.09)	-0.2	0.08 (0.02-0.17)	0.03 (0.01-0.06)	-3.14 (-3.46--2.83)
Angola	324.68 (66.01-617.04)	612.82 (136.88-1130.95)	0.89	6.59 (1.37-12.54)	3.85 (0.86-6.96)	-1.73 (-1.93--1.53)
Antigua and Barbuda	0.03 (0.01-0.05)	0.02 (0-0.03)	-0.33	0.06 (0.02-0.11)	0.02 (0-0.03)	-3.7 (-4.09--3.31)
Argentina	175.78 (39.01-309.07)	178.43 (40.27-319.55)	0.02	0.54 (0.12-0.96)	0.34 (0.08-0.61)	-1.25 (-1.6--0.9)
Armenia	7.21 (1.64-13.34)	4.91 (1.13-9.03)	-0.32	0.23 (0.05-0.42)	0.13 (0.03-0.24)	-2.06 (-3.06--1.05)
Australia	29.9 (6.58-53.23)	36.32 (8.36-64.91)	0.21	0.15 (0.03-0.27)	0.09 (0.02-0.15)	-1.76 (-2.13--1.4)
Austria	20.05 (4.46-37.28)	11.97 (2.66-21.69)	-0.4	0.18 (0.04-0.33)	0.07 (0.02-0.13)	-2.88 (-3.05--2.71)
Azerbaijan	76.89 (16.75-145.63)	83.71 (18.65-165.8)	0.09	1.31 (0.28-2.48)	0.68 (0.15-1.34)	-2.13 (-2.65--1.61)
Bahamas	0.54 (0.13-0.93)	0.45 (0.11-0.82)	-0.17	0.29 (0.07-0.5)	0.1 (0.02-0.18)	-3.41 (-3.8--3.02)
Bahrain	0.83	1.51	0.82	0.37	0.14	-3.6

	(0.19-1.5)	(0.36-2.78)		(0.09-0.67)	(0.04-0.27)	(-3.76--3.43)
Bangladesh	7037.33 (1459.11-12744.3)	6120.91 (1394.77-11397.77)	-0.13	12.67 (2.63-22.5)	4.08 (0.94-7.59)	-3.39 (-3.52--3.27)
Barbados	0.3 (0.07-0.55)	0.15 (0.04-0.27)	-0.5	0.12 (0.03-0.21)	0.03 (0.01-0.06)	-4.27 (-4.68--3.86)
Belarus	86.3 (17.68-165.68)	61.4 (11.78-119.13)	-0.29	0.69 (0.14-1.34)	0.45 (0.09-0.86)	-1.97 (-3.34--0.58)
Belgium	45.66 (10.17-82.26)	23.36 (5.42-42.73)	-0.49	0.31 (0.07-0.56)	0.11 (0.03-0.21)	-3.49 (-3.66--3.33)
Belize	0.33 (0.08-0.57)	0.6 (0.14-1.08)	0.82	0.32 (0.08-0.57)	0.16 (0.04-0.29)	-2.56 (-3--2.11)
Benin	69.41 (16.29-128.9)	121.73 (26.51-227.05)	0.75	3.27 (0.79-5.99)	1.99 (0.44-3.74)	-1.62 (-1.77--1.47)
Bermuda	0.02 (0-0.03)	0.01 (0-0.02)	-0.5	0.02 (0.01-0.04)	0.01 (0-0.03)	-1.62 (-2.14--1.09)
Bhutan	25.09 (4.84-52.08)	14.32 (2.91-31.42)	-0.43	8.53 (1.77-18.16)	2.15 (0.44-4.75)	-4.66 (-4.85--4.46)
Bolivia (Plurinational State of)	48.63 (10.28-89.86)	42.54 (9.03-75.66)	-0.13	1.34 (0.28-2.47)	0.42 (0.09-0.75)	-3.91 (-4.16--3.65)
Bosnia and Herzegovina	59.22 (13.26-109.91)	23.92 (5.18-45.48)	-0.6	1.37 (0.31-2.57)	0.43 (0.09-0.82)	-3.85 (-4.07--3.63)
Botswana	21.25 (4.65-41.47)	28.34 (6.55-55.22)	0.33	3.27 (0.73-6.33)	1.53 (0.35-2.97)	-2.64 (-3.13--2.14)
Brazil	475.88 (116.24-836.4)	853.4 (194.96-1504.03)	0.79	0.44 (0.11-0.78)	0.33 (0.08-0.58)	-0.82 (-1.01--0.63)
Brunei Darussalam	3.23 (0.7-6.1)	2.95 (0.67-5.44)	-0.09	2.55 (0.59-4.68)	0.8 (0.18-1.45)	-3.65 (-3.94--3.35)
Bulgaria	84.92 (19.94-154.51)	34.12 (8.5-63.79)	-0.6	0.7 (0.16-1.24)	0.3 (0.07-0.55)	-3.29 (-3.61--2.96)

Burkina Faso	146.99 (31.81-268.85)	334.65 (69.65-613.91)	1.28	3.29 (0.74-5.98)	3.12 (0.66-5.58)	0.09 (-0.05-0.23)
Burundi	183.79 (40.73-324)	206.06 (47.49-383.92)	0.12	7.12 (1.62-12.44)	3.55 (0.88-6.54)	-2.87 (-3.1--2.65)
Cabo Verde	7.23 (1.61-13.59)	10.11 (2.5-23.85)	0.4	3.32 (0.75-6.3)	1.98 (0.48-4.66)	-2.13 (-2.32--1.93)
Cambodia	72.91 (15.74-136.83)	90.33 (21.08-173.41)	0.24	1.53 (0.34-2.85)	0.69 (0.16-1.33)	-3.02 (-3.26--2.77)
Cameroon	266.16 (57-509.27)	642.71 (138.7-1343.08)	1.41	5.49 (1.2-10.24)	4.14 (0.94-8.9)	-0.76 (-1.06--0.46)
Canada	33.57 (7.9-62.78)	22.53 (5.2-40.54)	-0.33	0.11 (0.02-0.2)	0.04 (0.01-0.06)	-4 (-4.35--3.65)
Central African Republic	390.59 (86.4-728.73)	748.1 (175.82-1465.62)	0.92	26.18 (5.77-48.75)	23 (5.6-44.5)	-0.43 (-0.59--0.27)
Chad	224.81 (51.7-428.79)	445.54 (109.11-859.71)	0.98	7.67 (1.78-14.58)	6.52 (1.6-12.51)	-0.78 (-1.19--0.36)
Chile	238.43 (49.88-442.65)	208.16 (49.22-386.2)	-0.13	2.23 (0.47-4.1)	0.86 (0.2-1.59)	-2.81 (-2.98--2.63)
China	4793.07 (1159.1-8548.78)	3170.63 (834.53-5761.16)	-0.34	0.53 (0.13-0.93)	0.15 (0.04-0.28)	-3.72 (-3.88--3.56)
Colombia	52.5 (12.47-89.13)	64.14 (14.03-114.57)	0.22	0.26 (0.06-0.44)	0.12 (0.03-0.21)	-2.74 (-3.13--2.35)
Comoros	9.44 (2.01-17.93)	11.15 (2.52-21.76)	0.18	4.44 (0.95-8.27)	2.07 (0.48-4.08)	-2.88 (-3.17--2.59)
Congo	86.07 (17.17-156.93)	131.44 (31.03-273.4)	0.53	6.99 (1.44-12.65)	3.6 (0.87-7.21)	-2.45 (-2.66--2.24)
Cook Islands	0.06 (0.01-0.11)	0.04 (0.01-0.08)	-0.33	0.42 (0.1-0.76)	0.19 (0.04-0.34)	-2.44 (-2.66--2.23)
Costa Rica	6.03 (1.39-10.4	4.81 (1.03-8.58)	-0.2	0.32 (0.07-0.56)	0.09 (0.02-0.16)	-4.88 (-5.34-

	5)					-4.42)
C么te d'Ivoire	304.73 (55.99-613.62)	523.59 (125.87-1054.92)	0.72	6.27 (1.28-12.08)	3.57 (0.88-7)	-1.93 (-2.23--1.62)
Croatia	20.94 (5.23-37.14)	3.9 (0.89-7.09)	-0.81	0.34 (0.08-0.61)	0.05 (0.01-0.09)	-5.7 (-5.92--5.49)
Cuba	5.25 (1.18-9.12)	3.78 (0.97-6.97)	-0.28	0.05 (0.01-0.09)	0.02 (0.01-0.04)	-2.4 (-2.83--1.97)
Cyprus	2.08 (0.47-3.98)	0.95 (0.24-1.7)	-0.54	0.34 (0.07-0.64)	0.05 (0.01-0.1)	-6.2 (-6.65--5.75)
Czechia	43.6 (10.65-79.24)	10.14 (2.46-19.18)	-0.77	0.33 (0.08-0.59)	0.06 (0.01-0.11)	-5.14 (-5.53--4.74)
Democratic People's Republic of Korea	434.99 (101.14-816.17)	556.87 (117.19-999.98)	0.28	2.47 (0.57-4.63)	1.64 (0.35-2.95)	-1.45 (-1.73--1.17)
Democratic Republic of the Congo	4316.8 (930.81-8603.34)	5770.17 (1204.04-11674.85)	0.34	22.13 (4.95-42.98)	11.45 (2.4-22.71)	-2.25 (-2.38--2.12)
Denmark	11.9 (2.4-22.51)	6.62 (1.42-12.44)	-0.44	0.16 (0.03-0.3)	0.06 (0.01-0.12)	-3.28 (-3.56--3)
Djibouti	4.57 (1.05-9.26)	15.85 (3.79-31.58)	2.47	2.71 (0.66-5.29)	2.09 (0.49-4.09)	-0.97 (-1.36--0.58)
Dominica	0.23 (0.05-0.4)	0.19 (0.04-0.35)	-0.17	0.4 (0.09-0.7)	0.24 (0.06-0.44)	-1.54 (-1.89--1.19)
Dominican Republic	22.33 (5.15-40.28)	43.89 (9.74-86.69)	0.97	0.51 (0.12-0.92)	0.41 (0.09-0.81)	0.28 (-0.26-0.83)
Ecuador	103.79 (23.37-192.75)	42.24 (9.67-76.41)	-0.59	1.67 (0.4-3.05)	0.25 (0.06-0.44)	-5.5 (-5.82--5.18)
Egypt	40.87 (9.35-73.65)	65.17 (15.16-120.41)	0.59	0.14 (0.03-0.24)	0.09 (0.02-0.16)	-0.76 (-1.03--0.5)
El Salvador	27.08 (6.02-48.69)	14.54 (3.64-25.76)	-0.46	0.84 (0.19-1.49)	0.24 (0.06-0.42)	-4.29 (-4.75--3.83)

Equatorial Guinea	31.78 (6.63-60.68)	49.63 (11.05-106.11)	0.56	14.05 (2.98-26.8)	6.03 (1.28-12.18)	-2.66 (-3.15--2.16)
Eritrea	86.93 (17.87-165.1)	145.73 (30.29-289.73)	0.68	5.86 (1.27-10.97)	4.08 (0.87-7.72)	-1.37 (-1.55--1.18)
Estonia	54.52 (8.71-112.93)	22.42 (4.2-44.28)	-0.59	2.89 (0.46-5.99)	1.2 (0.22-2.42)	-3.78 (-5.16--2.38)
Eswatini	11.16 (2.37-21.15)	27.33 (5.89-52.28)	1.45	3.23 (0.71-6.09)	3.8 (0.85-7.17)	1.11 (0.21-2.01)
Ethiopia	2208.05 (497.45-3950.65)	915.24 (217.54-1707.55)	-0.59	9.62 (2.22-17.15)	1.89 (0.46-3.5)	-5.83 (-6.07--5.58)
Fiji	3.69 (0.83-6.8)	5.32 (1.25-9.77)	0.44	0.82 (0.19-1.49)	0.64 (0.15-1.16)	-0.95 (-1.11--0.8)
Finland	49.99 (11.14-91.32)	11.98 (2.89-21.56)	-0.76	0.71 (0.16-1.3)	0.11 (0.03-0.2)	-6.05 (-6.57--5.52)
France	548.19 (114.49-995.77)	196.85 (44.13-360.83)	-0.64	0.69 (0.14-1.24)	0.16 (0.03-0.29)	-5.31 (-5.55--5.07)
Gabon	48.54 (9.79-96.8)	81.57 (17.73-158.85)	0.68	7.83 (1.57-15.69)	6.04 (1.31-11.69)	-0.94 (-1.35--0.53)
Gambia	24.03 (5.12-46.9)	54.9 (11.91-107.86)	1.28	6.01 (1.26-11.78)	4.77 (1.06-9.16)	-0.85 (-1.03--0.68)
Georgia	40 (8.9-73.17)	16.64 (4.25-29.64)	-0.58	0.65 (0.14-1.18)	0.34 (0.09-0.6)	-1.36 (-2.02--0.69)
Germany	565.56 (132.17-1027.5)	151.51 (33.62-272.01)	-0.73	0.48 (0.11-0.87)	0.09 (0.02-0.17)	-5.13 (-5.41--4.85)
Ghana	430.9 (103.76-746.28)	1431.89 (316.1-2739.42)	2.32	6.53 (1.57-11.44)	7.84 (1.77-14.87)	1.11 (0.93-1.28)
Greece	38.45 (9.88-68.83)	18.93 (4.38-33.82)	-0.51	0.26 (0.07-0.46)	0.09 (0.02-0.15)	-3.24 (-3.81--2.67)
Greenland	0.72 (0.15-1.42)	0.73 (0.17-1.37)	0.01	2.12 (0.47-4.06)	1.07 (0.25-2)	-2.6 (-2.95-

						-2.26)
Grenada	0.05 (0.01-0.09)	0.03 (0.01-0.05)	-0.4	0.08 (0.02-0.14)	0.02 (0.01-0.04)	-3.39 (-3.75- -3.02)
Guam	0.82 (0.2-1.46)	1.13 (0.29-2.02)	0.38	0.88 (0.21-1.58)	0.6 (0.15-1.07)	-1.53 (-1.82- -1.23)
Guatemala	59.24 (13.03-105.84)	28.13 (6.76-50.69)	-0.53	1.39 (0.32-2.47)	0.22 (0.05-0.4)	-6.38 (-7.18- -5.57)
Guinea	177.71 (39.45-323.85)	256.32 (59.91-502.15)	0.44	5.14 (1.17-9.44)	3.96 (0.92-7.58)	-0.59 (-0.8-- 0.37)
Guinea-Bissau	53.65 (11.11-100.23)	65.77 (13.72-123.51)	0.23	11.83 (2.54-21.53)	7.06 (1.48-12.85)	-1.6 (-1.77- -1.43)
Guyana	7.86 (1.99-13.91)	6.02 (1.32-11.6)	-0.23	1.74 (0.45-3.11)	0.82 (0.18-1.58)	-1.68 (-1.99- -1.36)
Haiti	49.6 (9.8-186.56)	55.37 (10.95-206.87)	0.12	1.27 (0.25-5)	0.58 (0.11-2.21)	-2.23 (-2.45- -2.01)
Honduras	26.25 (5.95-47.36)	37.23 (8.36-69.26)	0.42	1.08 (0.25-1.95)	0.51 (0.12-0.95)	-2.48 (-2.59- -2.37)
Hungary	115.28 (28.41-196.67)	18.53 (3.93-34.54)	-0.84	0.81 (0.2-1.39)	0.12 (0.02-0.22)	-6.19 (-6.46- -5.91)
Iceland	1.07 (0.22-1.96)	0.59 (0.14-1.05)	-0.45	0.38 (0.08-0.69)	0.11 (0.03-0.19)	-4.41 (-4.68- -4.13)
India	11709.8 (2994.02-19933.72)	11350.83 (2847.71-19894.22)	-0.03	2.23 (0.58-3.79)	0.88 (0.22-1.55)	-3.16 (-3.33- -2.99)
Indonesia	1129.18 (259.41-2002.26)	1530.8 (370.62-2694.31)	0.36	1.09 (0.26-1.9)	0.61 (0.15-1.07)	-1.81 (-1.93- -1.68)
Iran (Islamic Republic of)	30.55 (6.87-55.79)	42.51 (10.81-78.26)	0.39	0.11 (0.03-0.2)	0.05 (0.01-0.09)	-2.53 (-2.7-- 2.35)
Iraq	122.09 (28.43-228.86)	85.12 (20.38-165.74)	-0.3	1.33 (0.31-2.52)	0.27 (0.07-0.51)	-5.35 (-5.48- -5.21)
Ireland	14.87	6.68	-0.55	0.37	0.09	-4.83

	(3.43-27.52)	(1.53-12.28)		(0.08-0.69)	(0.02-0.16)	(-5.13--4.52)
Israel	7.12 (1.53-12.5)	4.42 (1.03-7.89)	-0.38	0.15 (0.03-0.26)	0.04 (0.01-0.07)	-5.41 (-5.76--5.07)
Italy	262.88 (58.9-474.27)	98.66 (23.23-182.3)	-0.62	0.3 (0.07-0.55)	0.08 (0.02-0.14)	-4.57 (-4.79--4.34)
Jamaica	0.63 (0.15-1.08)	0.44 (0.1-0.79)	-0.3	0.04 (0.01-0.06)	0.01 (0-0.03)	-3.08 (-3.41--2.75)
Japan	2136.35 (513.05-3794.85)	786.61 (198.42-1371.49)	-0.63	1.23 (0.3-2.19)	0.2 (0.05-0.35)	-6.54 (-7.1--5.97)
Jordan	2.6 (0.63-4.83)	4.78 (1.19-9.13)	0.84	0.17 (0.04-0.31)	0.05 (0.01-0.1)	-4.21 (-4.42--4)
Kazakhstan	201.18 (44.43-370.9)	113.99 (25.44-212.88)	-0.43	1.39 (0.3-2.58)	0.56 (0.13-1.04)	-5.29 (-6.88--3.67)
Kenya	323.89 (66.3-647.3)	1002.68 (254.53-1960.11)	2.1	3.57 (0.75-7.29)	3.71 (0.95-7.21)	0.55 (0.17-0.94)
Kiribati	5.74 (1.38-10.38)	7.75 (2-14.12)	0.35	12.45 (3.06-22.71)	8.57 (2.29-15.37)	-1.11 (-1.18--1.03)
Kuwait	1.61 (0.39-2.84)	4.18 (1.01-7.53)	1.6	0.17 (0.04-0.3)	0.12 (0.03-0.21)	-1.75 (-2.24--1.26)
Kyrgyzstan	36.13 (7.56-69.31)	55.03 (10.76-105.4)	0.52	1.1 (0.23-2.07)	0.9 (0.18-1.7)	-1.81 (-2.97--0.64)
Lao People's Democratic Republic	46.98 (10.22-85.31)	31.45 (7.08-59.29)	-0.33	2.09 (0.45-3.78)	0.61 (0.14-1.14)	-4.37 (-4.6--4.14)
Latvia	142 (21.51-297.56)	60.73 (11.2-124.75)	-0.57	4.3 (0.64-9.21)	2.25 (0.39-4.74)	-2.9 (-4.05--1.75)
Lebanon	4.86 (1.07-9.01)	3.35 (0.78-6)	-0.31	0.21 (0.05-0.39)	0.06 (0.01-0.1)	-4.38 (-4.59--4.16)
Lesotho	28.35 (7.13-53.73)	99.28 (23.29-191.08)	2.5	3.1 (0.79-5.86)	7.73 (1.81-14.78)	4.08 (3.43-4.73)

Liberia	79.45 (18.4-147.61)	112.88 (26.87-247.51)	0.42	6.4 (1.52-11.76)	3.71 (0.84-7.89)	-1.8 (-1.99--1.61)
Libya	4.27 (0.86-7.93)	8.43 (2.16-16.44)	0.97	0.2 (0.04-0.37)	0.13 (0.03-0.25)	-0.91 (-1.14--0.68)
Lithuania	240.68 (37.94-503.82)	129.39 (22.3-266.26)	-0.46	5.72 (0.89-12.09)	3.35 (0.56-6.99)	-2.06 (-3.3--0.81)
Luxembourg	2.25 (0.43-4.39)	0.74 (0.15-1.4)	-0.67	0.43 (0.08-0.83)	0.08 (0.02-0.14)	-5.91 (-6.31--5.51)
Madagascar	228.55 (50.92-441.19)	364.04 (74.71-701.81)	0.59	3.96 (0.91-7.42)	2.66 (0.57-5.1)	-1.25 (-1.3--1.21)
Malawi	152.38 (32.33-293)	228.3 (51.38-457.43)	0.5	3.6 (0.79-6.68)	2.66 (0.61-5.27)	-1.33 (-1.64--1.02)
Malaysia	167.78 (37.58-294.02)	248.58 (59.47-436.07)	0.48	1.7 (0.38-2.92)	0.81 (0.19-1.41)	-2.48 (-2.85--2.11)
Maldives	1.14 (0.26-2.06)	0.43 (0.1-0.78)	-0.62	1.14 (0.24-2.08)	0.12 (0.03-0.2)	-7.8 (-8.22--7.38)
Mali	878.37 (223.63-1642.8)	1113.68 (238.67-2167.41)	0.27	18.98 (4.9-36.05)	10.25 (2.26-19.81)	-1.9 (-2.02--1.79)
Malta	1 (0.2-1.89)	0.42 (0.09-0.79)	-0.58	0.23 (0.05-0.44)	0.06 (0.01-0.11)	-4.89 (-5.28--4.49)
Marshall Islands	2.42 (0.63-4.7)	3.15 (0.73-6.12)	0.3	10.69 (2.75-20.34)	6.86 (1.57-13.24)	-1.38 (-1.53--1.23)
Mauritania	50.98 (11.24-93.81)	46.54 (11.34-90.59)	-0.09	4.87 (1.09-8.95)	2.02 (0.5-3.9)	-2.93 (-3.01--2.85)
Mauritius	1.51 (0.38-2.61)	1.06 (0.23-1.9)	-0.3	0.17 (0.04-0.3)	0.06 (0.01-0.11)	-3.22 (-3.47--2.97)
Mexico	859.81 (207.58-1532.79)	446.8 (108.69-813.78)	-0.48	1.66 (0.4-2.89)	0.32 (0.08-0.59)	-5.26 (-5.72--4.8)
Micronesia (Federated	1.48 (0.31-2.87)	0.91 (0.22-1.7)	-0.39	2.63 (0.56-5.05)	0.99 (0.23-1.87)	-3.44 (-3.54-

States of)						-3.35)
Monaco	0.62 (0.12-1.19)	0.32 (0.06-0.61)	-0.48	1 (0.2-1.91)	0.4 (0.07-0.76)	-3.09 (-3.41--2.77)
Mongolia	47.6 (8.59-104.12)	105.37 (21.84-235.48)	1.21	3.74 (0.71-8.06)	3.13 (0.67-7.03)	-1.04 (-1.32--0.77)
Montenegro	4.65 (0.87-9.28)	3.26 (0.73-6.46)	-0.3	0.71 (0.13-1.41)	0.36 (0.08-0.71)	-2.33 (-2.79--1.88)
Morocco	223.86 (46.08-598.53)	275.95 (66.67-604.01)	0.23	1.46 (0.3-4.07)	0.74 (0.18-1.6)	-1.71 (-1.99--1.42)
Mozambique	239.82 (57.89-472.14)	538.81 (115.32-1011.09)	1.25	3.65 (0.89-7.09)	3.93 (0.87-7.29)	1.09 (0.77-1.4)
Myanmar	661.1 (168.77-1184.64)	384.36 (88.48-691.59)	-0.42	2.58 (0.66-4.6)	0.73 (0.17-1.31)	-4.48 (-4.78--4.19)
Namibia	33.95 (7.05-65.01)	47.47 (10.46-92.2)	0.4	4.59 (0.98-8.7)	2.81 (0.61-5.52)	-1.91 (-2.46--1.35)
Nauru	0.22 (0.05-0.42)	0.16 (0.04-0.31)	-0.27	3.56 (0.78-6.84)	2.08 (0.47-4.03)	-1.86 (-2.05--1.67)
Nepal	1596.22 (335.91-2929.03)	1333.63 (320.08-2604.8)	-0.16	15.13 (3.21-27.98)	5.32 (1.28-10.39)	-3.59 (-3.7--3.49)
Netherlands	34.49 (7.81-61.33)	17.07 (3.99-30.38)	-0.51	0.18 (0.04-0.31)	0.05 (0.01-0.09)	-4.34 (-4.55--4.13)
New Zealand	11.43 (2.64-20.82)	3.93 (0.91-7.21)	-0.66	0.3 (0.07-0.55)	0.05 (0.01-0.1)	-5.39 (-6.04--4.73)
Nicaragua	12.93 (3.06-22.72)	12.23 (2.88-22.18)	-0.05	0.71 (0.17-1.24)	0.22 (0.05-0.39)	-4.15 (-4.29--4.01)
Niger	393.71 (83.48-763.35)	695.65 (160.99-1386.3)	0.77	11.17 (2.39-21.36)	6.73 (1.55-13.5)	-1.86 (-2.05--1.67)
Nigeria	2452.39 (553.45-4462.62)	3212.98 (748.83-5695.45)	0.31	5.14 (1.18-9.21)	3.02 (0.72-5.47)	-2.05 (-2.43--1.68)
Niue	0.02	0.01	-0.5	0.86	0.57	-1.46

	(0-0.03)	(0-0.02)		(0.2-1.6)	(0.15-1.03)	(-1.56--1.35)
North Macedonia	21.89 (4.65-40.77)	8.66 (1.99-16.76)	-0.6	1.11 (0.24-2.08)	0.28 (0.07-0.54)	-4.31 (-4.64--3.97)
Northern Mariana Islands	0.96 (0.22-1.82)	0.46 (0.12-0.85)	-0.52	3.07 (0.67-5.72)	0.84 (0.23-1.52)	-4.31 (-4.58--4.04)
Norway	69.38 (14.34-128.3)	37.24 (8.25-68.45)	-0.46	1 (0.2-1.87)	0.37 (0.08-0.68)	-3.3 (-3.63--2.97)
Oman	1.29 (0.25-2.44)	1.55 (0.34-3.11)	0.2	0.16 (0.03-0.31)	0.05 (0.01-0.1)	-2.96 (-3.32--2.6)
Pakistan	11148.04 (2562.13-20918.66)	16518.76 (3695.76-31886.3)	0.48	18.01 (4.11-34.33)	10.51 (2.41-20.39)	-2.02 (-2.47--1.58)
Palau	0.13 (0.03-0.25)	0.17 (0.04-0.3)	0.31	1.14 (0.26-2.11)	0.72 (0.19-1.29)	-1.27 (-1.38--1.17)
Palestine	1.13 (0.23-2.1)	1.15 (0.3-2.15)	0.02	0.13 (0.03-0.23)	0.04 (0.01-0.07)	-3.82 (-3.98--3.66)
Panama	10.25 (2.39-17.72)	10.94 (2.39-19.29)	0.07	0.63 (0.15-1.11)	0.25 (0.05-0.44)	-3.05 (-3.4--2.7)
Papua New Guinea	58.16 (14.03-114.15)	95.35 (19.72-175.73)	0.64	2.58 (0.64-4.94)	1.44 (0.3-2.61)	-1.92 (-1.97--1.88)
Paraguay	10.61 (2.32-18.93)	20.83 (4.76-38.52)	0.96	0.43 (0.1-0.77)	0.32 (0.07-0.6)	-0.78 (-0.88--0.69)
Peru	58.56 (12.79-103.81)	50.94 (11.51-94.37)	-0.13	0.45 (0.1-0.78)	0.15 (0.03-0.27)	-3.97 (-4.74--3.19)
Philippines	464.01 (111.66-833.95)	1187.88 (272.35-2124.69)	1.56	1.33 (0.32-2.36)	1.3 (0.3-2.31)	0.33 (0.16-0.5)
Poland	287.65 (70.33-513.51)	130.47 (32.69-239.47)	-0.55	0.66 (0.16-1.18)	0.22 (0.06-0.41)	-3.28 (-3.56--3)
Portugal	74.96 (16.48-132.53)	34.99 (8.58-63.98)	-0.53	0.57 (0.12-1)	0.16 (0.04-0.3)	-4.58 (-4.93--4.24)

Puerto Rico	7.95 (2.07-14.26)	1.99 (0.49-3.61)	-0.75	0.22 (0.06-0.4)	0.04 (0.01-0.08)	-5.09 (-5.36--4.82)
Qatar	1.37 (0.31-2.53)	3.45 (0.73-6.64)	1.52	0.89 (0.21-1.67)	0.3 (0.07-0.56)	-4.02 (-4.46--3.57)
Republic of Korea	1006.44 (224.09-1792.51)	381.59 (86.28-670.99)	-0.62	3.14 (0.69-5.65)	0.42 (0.1-0.74)	-6.35 (-6.54--6.15)
Republic of Moldova	64.77 (12.95-129.36)	57.14 (13.01-108.81)	-0.12	1.39 (0.28-2.79)	1.09 (0.25-2.09)	-0.99 (-2.26-0.29)
Romania	288.86 (58.97-550.03)	469.9 (109.13-888.71)	0.63	1.06 (0.22-2.06)	1.63 (0.37-3.01)	1.04 (0.41-1.68)
Russian Federation	11901.14 (1928-25007.82)	2250.48 (482.05-4240.05)	-0.81	6.68 (1.06-13.98)	1.11 (0.24-2.09)	-7.46 (-9.14--5.75)
Rwanda	147.31 (32.41-271.17)	88.59 (20.79-168.8)	-0.4	4.59 (1.04-8.27)	1.24 (0.29-2.39)	-5.37 (-5.88--4.86)
Saint Kitts and Nevis	0.1 (0.02-0.18)	0.05 (0.01-0.1)	-0.5	0.3 (0.07-0.54)	0.07 (0.02-0.13)	-4.88 (-5.5--4.26)
Saint Lucia	0.33 (0.08-0.56)	0.24 (0.06-0.45)	-0.27	0.36 (0.09-0.62)	0.11 (0.02-0.2)	-4.08 (-4.45--3.71)
Saint Vincent and the Grenadines	0.2 (0.05-0.35)	0.12 (0.03-0.21)	-0.4	0.27 (0.06-0.47)	0.09 (0.02-0.16)	-3.11 (-3.45--2.77)
Samoa	1.21 (0.29-2.34)	1.52 (0.41-2.8)	0.26	1.24 (0.3-2.38)	0.95 (0.25-1.72)	-0.57 (-0.89--0.26)
San Marino	0.06 (0.01-0.12)	0.05 (0.01-0.09)	-0.17	0.19 (0.04-0.35)	0.07 (0.02-0.14)	-3.32 (-3.51--3.13)
Sao Tome and Principe	1.49 (0.34-2.83)	2.19 (0.54-4.34)	0.47	2.25 (0.51-4.26)	1.68 (0.43-3.29)	-1.25 (-1.52--0.99)
Saudi Arabia	73.76 (14.75-133.96)	177.23 (43.79-341.38)	1.4	0.94 (0.19-1.69)	0.44 (0.11-0.86)	-2.78 (-2.99--2.57)
Senegal	315.52 (68.54-565	395.59 (94.92-742	0.25	8.89 (1.95-15.76)	4.48 (1.07-8.31)	-2.1 (-2.23-

	.87)	.52)				-1.97)
Serbia	65.74 (14.35-124.88)	20.22 (4.88-37.53)	-0.69	0.56 (0.12-1.05)	0.14 (0.04-0.26)	-5.04 (-5.37--4.71)
Seychelles	0.25 (0.06-0.47)	0.23 (0.06-0.41)	-0.08	0.45 (0.11-0.83)	0.18 (0.04-0.32)	-3.68 (-3.98--3.38)
Sierra Leone	101.54 (22.58-183.98)	161.18 (34.94-311.17)	0.59	4.72 (1.07-8.49)	3.58 (0.79-6.95)	-0.49 (-0.87--0.1)
Singapore	17.62 (4.29-31.44)	9.3 (2.18-16.64)	-0.47	0.76 (0.19-1.36)	0.11 (0.03-0.19)	-6.01 (-6.16--5.86)
Slovakia	26.44 (6.22-48.8)	14.31 (3.01-26.73)	-0.46	0.45 (0.11-0.83)	0.16 (0.03-0.31)	-2.9 (-3.01--2.78)
Slovenia	26.31 (5.21-52.45)	6.96 (1.56-12.85)	-0.74	1.08 (0.21-2.15)	0.19 (0.04-0.36)	-5.47 (-6.27--4.66)
Solomon Islands	1.61 (0.35-3.03)	2.64 (0.65-4.9)	0.64	0.96 (0.21-1.84)	0.56 (0.14-1.02)	-1.8 (-1.99--1.62)
Somalia	216.68 (45.14-458.87)	536.66 (131.11-1067.78)	1.48	7.24 (1.6-14.53)	7.13 (1.75-13.89)	-0.14 (-0.26--0.02)
South Africa	587.03 (127.23-1061.66)	1325.08 (314.62-2280.82)	1.26	2.27 (0.5-4.08)	2.44 (0.58-4.19)	0.73 (-0.19-1.66)
South Sudan	123.07 (28.22-242.28)	201.31 (46.97-391.48)	0.64	4.47 (1.02-8.74)	4.42 (1.06-8.45)	-0.25 (-0.46--0.04)
Spain	496.26 (103.65-912.07)	89.24 (20.48-166.06)	-0.82	1 (0.2-1.83)	0.1 (0.02-0.19)	-7.25 (-7.69--6.81)
Sri Lanka	33.37 (7.68-59.05)	27.87 (7.11-51.09)	-0.16	0.28 (0.06-0.49)	0.1 (0.03-0.19)	-3.39 (-3.77--3.01)
Sudan	57.46 (11.65-107.35)	48.92 (11.05-94.84)	-0.15	0.57 (0.12-1.07)	0.2 (0.05-0.4)	-3.27 (-3.38--3.16)
Suriname	1.01 (0.23-1.79)	1.05 (0.28-2.02)	0.04	0.34 (0.08-0.59)	0.17 (0.04-0.32)	-2.45 (-2.8--2.1)
Sweden	51.2	22.28	-0.56	0.32	0.11	-3.79

	(10.29-94.04)	(4.74-43.25)		(0.06-0.6)	(0.02-0.21)	(-4.11--3.48)
Switzerland	22.36 (4.74-40.35)	6.5 (1.51-11.74)	-0.71	0.23 (0.05-0.42)	0.04 (0.01-0.07)	-5.47 (-5.66--5.28)
Syrian Arab Republic	8.17 (2.02-14.74)	6.69 (1.73-12.85)	-0.18	0.14 (0.03-0.25)	0.05 (0.01-0.09)	-3.83 (-4.16--3.5)
Taiwan	183.13 (43.9-320.1)	76.54 (18.23-132.33)	-0.58	1.15 (0.27-2.02)	0.19 (0.04-0.33)	-6.33 (-6.6--6.06)
Tajikistan	73.12 (15.58-140.04)	102.66 (22.9-187.52)	0.4	2.28 (0.5-4.25)	1.28 (0.29-2.3)	-2.48 (-2.87--2.09)
Thailand	214.18 (53.52-390.54)	200.31 (46.35-369.06)	-0.06	0.61 (0.15-1.09)	0.19 (0.04-0.35)	-3.97 (-4.13--3.81)
Timor-Leste	4.22 (0.89-8.04)	7.46 (1.67-14.08)	0.77	1.36 (0.31-2.55)	0.85 (0.19-1.59)	-1.79 (-1.96--1.62)
Togo	57.55 (12.94-109.57)	148.29 (33.95-289.78)	1.58	4.27 (0.97-7.87)	3.4 (0.81-6.56)	-0.65 (-0.96--0.33)
Tokelau	0.02 (0-0.04)	0.01 (0-0.01)	-0.5	1.49 (0.32-2.93)	0.6 (0.15-1.06)	-3.01 (-3.16--2.86)
Tonga	0.4 (0.1-0.77)	0.38 (0.1-0.7)	-0.05	0.65 (0.16-1.26)	0.45 (0.12-0.83)	-1.35 (-1.52--1.17)
Trinidad and Tobago	1.67 (0.39-2.99)	1.3 (0.31-2.44)	-0.22	0.18 (0.04-0.32)	0.07 (0.02-0.13)	-3.16 (-3.48--2.83)
Tunisia	10.68 (2.4-20.14)	11.35 (2.55-22.95)	0.06	0.2 (0.05-0.37)	0.08 (0.02-0.17)	-2.89 (-3.02--2.75)
T 眉 rkiye	125.15 (26.73-242.18)	69.21 (17.05-126.92)	-0.45	0.34 (0.07-0.65)	0.07 (0.02-0.13)	-5.51 (-6.03--4.99)
Turkmenistan	32.73 (7.15-60.21)	95 (20.74-180.9)	1.9	1.39 (0.31-2.5)	1.86 (0.41-3.51)	0.16 (-0.53-0.86)
Tuvalu	0.26 (0.06-0.49)	0.14 (0.03-0.25)	-0.46	3.52 (0.84-6.51)	1.26 (0.29-2.23)	-3.24 (-3.33--3.14)

Uganda	223.88 (49-461.29)	396.11 (86.32-764.52)	0.77	3.17 (0.69-6.48)	2.18 (0.49-4.23)	-1.88 (-2.37--1.39)
Ukraine	257.34 (55.82-473.51)	364.38 (82.55-680.61)	0.42	0.37 (0.08-0.68)	0.57 (0.13-1.07)	1.47 (0.41-2.55)
United Arab Emirates	3.76 (0.72-8.8)	8.55 (1.76-17.2)	1.27	0.46 (0.09-0.96)	0.16 (0.03-0.31)	-3.43 (-3.6--3.25)
United Kingdom	199.77 (44.08-362.13)	106.58 (25-193.63)	-0.47	0.24 (0.05-0.44)	0.1 (0.02-0.18)	-2.57 (-2.82--2.31)
United Republic of Tanzania	353.59 (74.79-693.48)	546.17 (115.33-1031.25)	0.54	2.99 (0.64-5.65)	1.86 (0.41-3.45)	-1.65 (-1.79--1.52)
United States of America	1580.72 (350.53-2874.37)	994.87 (235.87-1791.5)	-0.37	0.53 (0.12-0.97)	0.21 (0.05-0.39)	-3.48 (-3.83--3.13)
United States Virgin Islands	0.11 (0.03-0.2)	0.09 (0.02-0.17)	-0.18	0.11 (0.02-0.19)	0.07 (0.02-0.13)	-1.44 (-1.61--1.26)
Uruguay	18.67 (4.29-35.07)	14.87 (3.24-28.27)	-0.2	0.51 (0.12-0.96)	0.33 (0.07-0.63)	-1.28 (-1.65--0.91)
Uzbekistan	129.24 (26.7-239.76)	241.32 (51.76-434.01)	0.87	0.99 (0.2-1.82)	0.72 (0.15-1.29)	-1.36 (-2.24--0.48)
Vanuatu	1.21 (0.24-2.64)	2.35 (0.53-4.52)	0.94	1.58 (0.31-3.41)	1.08 (0.24-2.06)	-1.24 (-1.36--1.11)
Venezuela (Bolivarian Republic of)	54.26 (12.5-91.68)	73.62 (16.6-133.8)	0.36	0.5 (0.12-0.85)	0.24 (0.05-0.43)	-2.7 (-3.31--2.08)
Viet Nam	223.9 (52.81-413.32)	205.7 (45.71-380.52)	-0.08	0.56 (0.13-1.04)	0.21 (0.05-0.37)	-3.12 (-3.19--3.05)
Yemen	19.35 (3.52-38.63)	25.13 (5.06-48.68)	0.3	0.36 (0.07-0.73)	0.16 (0.03-0.31)	-2.93 (-3.16--2.69)
Zambia	227.18 (49.22-394.72)	149.96 (31.25-303.58)	-0.34	6.85 (1.54-12.07)	1.78 (0.39-3.45)	-5.59 (-6.46--4.72)
Zimbabwe	105.5 (22.81-188	345.03 (82.54-654	2.27	2.31 (0.5-4.09)	3.89 (0.94-7.16)	2.73 (1.92-

.24)	.07)		3.54)

Supplementary table 1. The DALYs of TB attributable to DHPM cases and rates in 1990 and 2021 across 204 countries, and the trends from 1990 to 2021.

Location	1990_death cases (95% UI)	2021_death cases (95% UI)	Percentage change	1990_ASMR_per 100000(95% UI)	2021_ASMR_per 100 000(95% UI)	EAPC (95% CI)
Afghanistan	10.62 (2.39-22.91)	13.46 (3.22-27.09)	0.27	0.16 (0.03-0.34)	0.11 (0.03-0.23)	-1 (-1.09--0.9)
Albania	1.54 (0.31-3.22)	1.08 (0.24-2.31)	-0.3	0.08 (0.02-0.16)	0.03 (0.01-0.06)	-3.19 (-3.39--2.98)
Algeria	2.79 (0.59-5.19)	3.52 (0.84-6.94)	0.26	0.03 (0.01-0.05)	0.01 (0-0.02)	-2.89 (-3.06--2.73)
American Samoa	0 (0-0.01)	0 (0-0.01)	NA	0.01 (0-0.03)	0.01 (0-0.02)	-2 (-2.26--1.73)
Andorra	0 (0-0)	0 (0-0)	NA	0 (0-0.01)	0 (0-0)	-3.01 (-3.38--2.64)
Angola	9.21 (1.9-17.51)	16.29 (3.6-29.92)	0.77	0.22 (0.05-0.43)	0.13 (0.03-0.22)	-1.95 (-2.14--1.75)
Antigua and Barbuda	0 (0-0)	0 (0-0)	NA	0 (0-0)	0 (0-0)	-4.41 (-4.82--4)
Argentina	5.88 (1.34-10.37)	5.86 (1.33-10.42)	0	0.02 (0-0.03)	0.01 (0-0.02)	-1.51 (-1.85--1.16)
Armenia	0.18 (0.04-0.31)	0.12 (0.03-0.22)	-0.33	0.01 (0-0.01)	0 (0-0.01)	-2.12 (-3.18--1.04)
Australia	1.31 (0.3-2.32)	1.63 (0.37-2.93)	0.24	0.01 (0-0.01)	0 (0-0.01)	-2.15 (-2.51--1.78)
Austria	0.91 (0.2-1.7)	0.59 (0.14-1.08)	-0.35	0.01 (0-0.01)	0 (0-0.01)	-2.85 (-3--2.7)
Azerbaijan	1.94 (0.42-3.58)	1.95 (0.46-3.86)	0.01	0.03 (0.01-0.06)	0.02 (0-0.03)	-2.2 (-2.74--1.65)
Bahamas	0.01 (0-0.03)	0.01 (0-0.02)	0	0.01 (0-0.02)	0 (0-0)	-3.88 (-4.27--3.49)
Bahrain	0.02	0.04	1	0.02 (0-0.03)	0.01 (0-0.01)	-3.2

	(0.01-0.04)	(0.01-0.08)				(-3.35--3.06)
Bangladesh	210.98 (44.75-375.78)	182.62 (41.93-347.12)	-0.13	0.43 (0.09-0.78)	0.13 (0.03-0.25)	-3.69 (-3.88--3.49)
Barbados	0.01 (0-0.02)	0 (0-0.01)	-1	0 (0-0.01)	0 (0-0)	-4.53 (-4.96--4.1)
Belarus	2.28 (0.48-4.26)	1.63 (0.33-3.13)	-0.29	0.02 (0-0.03)	0.01 (0-0.02)	-2.09 (-3.41--0.75)
Belgium	1.99 (0.45-3.57)	1.09 (0.26-1.96)	-0.45	0.01 (0-0.02)	0 (0-0.01)	-3.72 (-3.93--3.51)
Belize	0.01 (0-0.02)	0.02 (0-0.03)	1	0.01 (0-0.02)	0 (0-0.01)	-3.11 (-3.56--2.65)
Benin	2.4 (0.58-4.35)	3.67 (0.81-7.12)	0.53	0.13 (0.03-0.23)	0.07 (0.02-0.14)	-1.83 (-1.97--1.68)
Bermuda	0 (0-0)	0 (0-0)	NA	0 (0-0)	0 (0-0)	-6.24 (-6.74--5.73)
Bhutan	0.75 (0.15-1.59)	0.46 (0.09-1.05)	-0.39	0.3 (0.07-0.64)	0.07 (0.01-0.17)	-4.75 (-4.94--4.55)
Bolivia (Plurinational State of)	1.58 (0.33-2.93)	1.35 (0.29-2.41)	-0.15	0.05 (0.01-0.09)	0.01 (0-0.03)	-4.05 (-4.28--3.83)
Bosnia and Herzegovina	2.07 (0.49-3.75)	0.9 (0.2-1.72)	-0.57	0.05 (0.01-0.1)	0.01 (0-0.03)	-4.27 (-4.55--4)
Botswana	0.65 (0.14-1.25)	0.83 (0.19-1.65)	0.28	0.11 (0.02-0.22)	0.05 (0.01-0.1)	-2.71 (-3.16--2.26)
Brazil	13.02 (3.19-22.54)	23.41 (5.47-40.46)	0.8	0.01 (0-0.02)	0.01 (0-0.02)	-1.16 (-1.31--1.01)
Brunei Darussalam	0.1 (0.02-0.19)	0.1 (0.02-0.18)	0	0.1 (0.02-0.18)	0.03 (0.01-0.06)	-3.14 (-3.45--2.83)
Bulgaria	2.8 (0.66-5.05)	1.07 (0.26-1.96)	-0.62	0.02 (0.01-0.04)	0.01 (0-0.02)	-4.02 (-4.36--3.68)

Burkina Faso	5.02 (1.13-9.02)	10.35 (2.21-18.37)	1.06	0.13 (0.03-0.24)	0.12 (0.03-0.21)	-0.2 (-0.35--0.05)
Burundi	5.94 (1.37-10.44)	6.33 (1.56-11.82)	0.07	0.27 (0.06-0.47)	0.14 (0.03-0.25)	-2.77 (-2.98--2.55)
Cabo Verde	0.24 (0.05-0.44)	0.29 (0.07-0.69)	0.21	0.11 (0.02-0.2)	0.06 (0.01-0.14)	-2.4 (-2.68--2.13)
Cambodia	2.45 (0.54-4.61)	2.98 (0.7-5.8)	0.22	0.06 (0.01-0.12)	0.03 (0.01-0.05)	-3.1 (-3.36--2.83)
Cameroon	8.71 (1.92-16.15)	18.66 (4.2-40.65)	1.14	0.21 (0.05-0.39)	0.15 (0.04-0.32)	-1.04 (-1.32--0.76)
Canada	1.37 (0.33-2.48)	0.93 (0.22-1.66)	-0.32	0 (0-0.01)	0 (0-0)	-4.32 (-4.65--3.99)
Central African Republic	10.61 (2.28-19.65)	19.13 (4.61-37.32)	0.8	0.83 (0.18-1.53)	0.72 (0.17-1.37)	-0.52 (-0.67--0.37)
Chad	8.06 (1.87-15)	13.71 (3.35-26.62)	0.7	0.3 (0.07-0.55)	0.24 (0.06-0.47)	-0.98 (-1.38--0.59)
Chile	7.94 (1.68-14.6)	7.3 (1.78-13.25)	-0.08	0.08 (0.02-0.14)	0.03 (0.01-0.05)	-2.96 (-3.18--2.74)
China	156.61 (38.79-273.29)	87.45 (21.91-163.3)	-0.44	0.02 (0.01-0.03)	0 (0-0.01)	-4.88 (-5.07--4.69)
Colombia	1.63 (0.39-2.79)	2.09 (0.47-3.73)	0.28	0.01 (0-0.02)	0 (0-0.01)	-2.99 (-3.34--2.63)
Comoros	0.31 (0.06-0.58)	0.37 (0.09-0.72)	0.19	0.18 (0.04-0.33)	0.08 (0.02-0.16)	-3.02 (-3.28--2.77)
Congo	2.59 (0.54-4.75)	3.5 (0.85-7.21)	0.35	0.24 (0.05-0.45)	0.12 (0.03-0.23)	-2.64 (-2.85--2.43)
Cook Islands	0 (0-0)	0 (0-0)	NA	0.01 (0-0.02)	0 (0-0.01)	-3.07 (-3.25--2.88)
Costa Rica	0.21 (0.05-0.36	0.15 (0.03-0.26	-0.29	0.01 (0-0.02)	0 (0-0)	-5.55 (-6.03-

	)	)				-5.07)
C 么 te d'Ivoire	8.87 (1.78-17.24)	14.47 (3.53-28.94)	0.63	0.23 (0.05-0.43)	0.12 (0.03-0.24)	-2.13 (-2.42--1.84)
Croatia	0.76 (0.19-1.35)	0.16 (0.04-0.29)	-0.79	0.01 (0-0.02)	0 (0-0)	-5.78 (-6--5.55)
Cuba	0.15 (0.04-0.27)	0.09 (0.02-0.16)	-0.4	0 (0-0)	0 (0-0)	-3.51 (-4.01--3.02)
Cyprus	0.09 (0.02-0.18)	0.04 (0.01-0.08)	-0.56	0.02 (0-0.04)	0 (0-0.01)	-6.46 (-7.04--5.88)
Czechia	1.55 (0.39-2.81)	0.34 (0.08-0.62)	-0.78	0.01 (0-0.02)	0 (0-0)	-5.55 (-5.97--5.13)
Democratic People's Republic of Korea	14.38 (3.37-27.3)	18.71 (4.08-33.85)	0.3	0.09 (0.02-0.17)	0.06 (0.01-0.1)	-1.68 (-1.95--1.4)
Democratic Republic of the Congo	118.86 (26.37-231.2)	149.25 (31.3-297.29)	0.26	0.72 (0.16-1.36)	0.36 (0.08-0.7)	-2.4 (-2.53--2.26)
Denmark	0.51 (0.11-0.94)	0.29 (0.07-0.53)	-0.43	0.01 (0-0.01)	0 (0-0)	-3.53 (-3.85--3.21)
Djibouti	0.13 (0.03-0.26)	0.48 (0.11-0.96)	2.69	0.1 (0.03-0.2)	0.08 (0.02-0.16)	-0.9 (-1.27--0.52)
Dominica	0.01 (0-0.01)	0.01 (0-0.01)	0	0.01 (0-0.03)	0.01 (0-0.01)	-1.94 (-2.35--1.53)
Dominican Republic	0.67 (0.16-1.2)	1.26 (0.29-2.5)	0.88	0.02 (0-0.03)	0.01 (0-0.02)	-0.17 (-0.73-0.38)
Ecuador	3 (0.73-5.37)	1.11 (0.26-1.97)	-0.63	0.05 (0.01-0.09)	0.01 (0-0.01)	-5.89 (-6.22--5.56)
Egypt	1.29 (0.3-2.29)	1.81 (0.43-3.26)	0.4	0.01 (0-0.01)	0 (0-0.01)	-1.23 (-1.52--0.93)
El Salvador	0.89 (0.21-1.58)	0.45 (0.12-0.8)	-0.49	0.03 (0.01-0.05)	0.01 (0-0.01)	-4.78 (-5.24--4.31)

Equatorial Guinea	0.95 (0.2-1.79)	1.15 (0.25-2.41)	0.21	0.49 (0.1-0.9)	0.18 (0.04-0.35)	-3.34 (-3.82--2.85)
Eritrea	2.32 (0.5-4.4)	4.02 (0.84-7.77)	0.73	0.21 (0.05-0.38)	0.15 (0.03-0.28)	-1.23 (-1.39--1.07)
Estonia	1.34 (0.23-2.74)	0.63 (0.12-1.21)	-0.53	0.07 (0.01-0.14)	0.03 (0.01-0.06)	-3.61 (-5--2.2)
Eswatini	0.33 (0.07-0.62)	0.78 (0.17-1.46)	1.36	0.11 (0.02-0.2)	0.13 (0.03-0.23)	0.98 (0.13-1.83)
Ethiopia	67.71 (15.71-120.7)	31.19 (7.51-57.5)	-0.54	0.36 (0.08-0.64)	0.08 (0.02-0.14)	-5.43 (-5.65--5.21)
Fiji	0.1 (0.02-0.18)	0.15 (0.04-0.28)	0.5	0.03 (0.01-0.05)	0.02 (0-0.04)	-0.9 (-1.1--0.69)
Finland	2.29 (0.51-4.22)	0.62 (0.15-1.14)	-0.73	0.03 (0.01-0.06)	0 (0-0.01)	-6.37 (-6.81--5.93)
France	27.55 (6.08-49.63)	11.14 (2.65-20.65)	-0.6	0.03 (0.01-0.06)	0.01 (0-0.01)	-5.81 (-6.13--5.49)
Gabon	1.46 (0.29-2.9)	2.09 (0.45-4.09)	0.43	0.25 (0.05-0.5)	0.18 (0.04-0.34)	-1.21 (-1.61--0.81)
Gambia	0.73 (0.15-1.45)	1.63 (0.37-3.18)	1.23	0.22 (0.05-0.43)	0.17 (0.04-0.33)	-1.01 (-1.18--0.85)
Georgia	1.04 (0.24-1.89)	0.46 (0.12-0.84)	-0.56	0.02 (0-0.03)	0.01 (0-0.02)	-1.3 (-1.95--0.65)
Germany	23.49 (5.66-43.41)	7.11 (1.62-12.74)	-0.7	0.02 (0-0.03)	0 (0-0.01)	-5.46 (-5.78--5.15)
Ghana	14.8 (3.51-26.11)	47.6 (10.69-91.47)	2.22	0.28 (0.07-0.5)	0.32 (0.08-0.61)	0.92 (0.77-1.07)
Greece	1.87 (0.48-3.34)	1.01 (0.24-1.84)	-0.46	0.01 (0-0.02)	0 (0-0.01)	-3.47 (-4.22--2.71)
Greenland	0.03 (0.01-0.05	0.03 (0.01-0.05	0	0.1 (0.02-0.18)	0.05 (0.01-0.09)	-2.58 (-2.96-

	)	)				-2.21)
Grenada	0 (0-0)	0 (0-0)	NA	0 (0-0)	0 (0-0)	-4.05 (-4.42--3.67)
Guam	0.02 (0.01-0.04)	0.03 (0.01-0.05)	0.5	0.03 (0.01-0.05)	0.02 (0-0.03)	-2.46 (-2.81--2.1)
Guatemala	1.73 (0.4-3.07)	0.78 (0.18-1.38)	-0.55	0.05 (0.01-0.08)	0.01 (0-0.01)	-6.81 (-7.62--5.98)
Guinea	6.19 (1.46-11.33)	8.03 (1.88-15.46)	0.3	0.2 (0.05-0.36)	0.15 (0.03-0.28)	-0.79 (-1.01--0.57)
Guinea-Bissau	1.66 (0.36-3)	1.88 (0.39-3.48)	0.13	0.44 (0.1-0.78)	0.27 (0.06-0.48)	-1.56 (-1.71--1.41)
Guyana	0.23 (0.06-0.41)	0.16 (0.04-0.32)	-0.3	0.06 (0.01-0.1)	0.02 (0.01-0.05)	-2.05 (-2.34--1.77)
Haiti	1.43 (0.29-5.86)	1.5 (0.28-5.91)	0.05	0.04 (0.01-0.18)	0.02 (0-0.08)	-2.36 (-2.56--2.15)
Honduras	0.79 (0.18-1.41)	1.15 (0.26-2.14)	0.46	0.04 (0.01-0.07)	0.02 (0-0.03)	-2.4 (-2.55--2.26)
Hungary	4.15 (1.08-7.11)	0.58 (0.13-1.05)	-0.86	0.03 (0.01-0.05)	0 (0-0.01)	-6.78 (-7--6.56)
Iceland	0.05 (0.01-0.09)	0.03 (0.01-0.05)	-0.4	0.02 (0-0.03)	0 (0-0.01)	-4.49 (-4.75--4.21)
India	370.47 (96.51-631.27)	358.55 (89.19-620.9)	-0.03	0.08 (0.02-0.14)	0.03 (0.01-0.05)	-3.34 (-3.51--3.16)
Indonesia	38.8 (9.21-67.7)	51.54 (12.77-91.2)	0.33	0.05 (0.01-0.08)	0.02 (0.01-0.04)	-1.85 (-1.99--1.7)
Iran (Islamic Republic of)	1.01 (0.24-1.81)	1.33 (0.34-2.46)	0.32	0 (0-0.01)	0 (0-0)	-2.92 (-3.1--2.73)
Iraq	3.59 (0.82-6.6)	2.21 (0.52-4.25)	-0.38	0.04 (0.01-0.08)	0.01 (0-0.02)	-5.55 (-5.73--5.38)
Ireland	0.68	0.31	-0.54	0.02 (0-0.03)	0 (0-0.01)	-5.1

	(0.16-1.23)	(0.07-0.55)				(-5.35--4.84)
Israel	0.32 (0.07-0.57)	0.2 (0.05-0.37)	-0.38	0.01 (0-0.01)	0 (0-0)	-5.93 (-6.32--5.54)
Italy	11.28 (2.54-20.22)	5.14 (1.27-9.3)	-0.54	0.01 (0-0.02)	0 (0-0.01)	-4.65 (-4.88--4.41)
Jamaica	0.02 (0.01-0.04)	0.01 (0-0.02)	-0.5	0 (0-0)	0 (0-0)	-3.38 (-3.73--3.02)
Japan	87.73 (21.37-153.71)	49.11 (12.52-85.76)	-0.44	0.05 (0.01-0.09)	0.01 (0-0.02)	-5.98 (-6.52--5.43)
Jordan	0.08 (0.02-0.15)	0.14 (0.03-0.26)	0.75	0.01 (0-0.01)	0 (0-0)	-4.13 (-4.32--3.94)
Kazakhstan	5.09 (1.14-9.5)	2.75 (0.63-5.09)	-0.46	0.04 (0.01-0.07)	0.01 (0-0.03)	-5.48 (-6.98--3.94)
Kenya	11.24 (2.38-23.01)	31.67 (7.83-61.44)	1.82	0.15 (0.03-0.31)	0.14 (0.04-0.27)	0.25 (-0.05-0.56)
Kiribati	0.16 (0.04-0.29)	0.21 (0.06-0.39)	0.31	0.39 (0.09-0.72)	0.28 (0.07-0.51)	-0.88 (-0.99--0.77)
Kuwait	0.04 (0.01-0.07)	0.13 (0.03-0.23)	2.25	0.01 (0-0.01)	0.01 (0-0.01)	-0.97 (-1.52--0.41)
Kyrgyzstan	0.91 (0.19-1.71)	1.36 (0.27-2.57)	0.49	0.03 (0.01-0.05)	0.02 (0-0.04)	-1.85 (-2.98--0.71)
Lao People's Democratic Republic	1.54 (0.32-2.79)	1 (0.23-1.9)	-0.35	0.08 (0.02-0.14)	0.02 (0.01-0.04)	-4.4 (-4.62--4.19)
Latvia	3.59 (0.57-7.3)	1.64 (0.32-3.23)	-0.54	0.1 (0.02-0.22)	0.05 (0.01-0.11)	-2.9 (-4.07--1.72)
Lebanon	0.16 (0.04-0.29)	0.11 (0.03-0.2)	-0.31	0.01 (0-0.01)	0 (0-0)	-4.77 (-4.94--4.59)
Lesotho	0.92 (0.23-1.77)	2.91 (0.68-5.56)	2.16	0.11 (0.03-0.2)	0.25 (0.06-0.47)	3.78 (3.14-4.43)

Liberia	2.54 (0.62-4.66)	2.87 (0.66-6.37)	0.13	0.23 (0.06-0.42)	0.12 (0.03-0.25)	-2.27 (-2.43--2.1)
Libya	0.14 (0.03-0.26)	0.25 (0.07-0.49)	0.79	0.01 (0-0.01)	0 (0-0.01)	-1.08 (-1.36--0.79)
Lithuania	5.93 (1-12)	3.5 (0.62-7.01)	-0.41	0.14 (0.02-0.28)	0.08 (0.01-0.16)	-2.03 (-3.28--0.76)
Luxembourg	0.09 (0.02-0.17)	0.03 (0.01-0.05)	-0.67	0.02 (0-0.03)	0 (0-0)	-6.31 (-6.71--5.9)
Madagascar	7.11 (1.62-13.6)	10.57 (2.24-20.73)	0.49	0.15 (0.03-0.28)	0.1 (0.02-0.19)	-1.27 (-1.32--1.21)
Malawi	4.91 (1.07-9.11)	7.26 (1.68-14.55)	0.48	0.14 (0.03-0.27)	0.11 (0.03-0.21)	-1.28 (-1.54--1.01)
Malaysia	5.65 (1.26-9.65)	7.21 (1.74-12.8)	0.28	0.06 (0.01-0.11)	0.03 (0.01-0.05)	-3.12 (-3.47--2.76)
Maldives	0.04 (0.01-0.07)	0.01 (0-0.02)	-0.75	0.04 (0.01-0.08)	0 (0-0.01)	-7.67 (-8.02--7.33)
Mali	26.2 (6.84-49.78)	31.52 (6.96-60.79)	0.2	0.65 (0.17-1.2)	0.35 (0.08-0.68)	-2 (-2.14--1.85)
Malta	0.04 (0.01-0.07)	0.01 (0-0.03)	-0.75	0.01 (0-0.02)	0 (0-0)	-6.02 (-6.48--5.57)
Marshall Islands	0.07 (0.02-0.13)	0.08 (0.02-0.16)	0.14	0.34 (0.09-0.65)	0.22 (0.05-0.43)	-1.33 (-1.47--1.2)
Mauritania	1.8 (0.41-3.31)	1.58 (0.4-3.1)	-0.12	0.19 (0.04-0.35)	0.08 (0.02-0.15)	-3.04 (-3.12--2.96)
Mauritius	0.04 (0.01-0.07)	0.02 (0.01-0.04)	-0.5	0 (0-0.01)	0 (0-0)	-3.84 (-4.15--3.53)
Mexico	24.37 (5.8-41.97)	11.03 (2.75-19.86)	-0.55	0.05 (0.01-0.09)	0.01 (0-0.01)	-6.05 (-6.5--5.6)
Micronesia (Federated	0.04 (0.01-0.08	0.02 (0.01-0.04	-0.5	0.08 (0.02-0.15)	0.03 (0.01-0.05)	-3.51 (-3.6--

States of)	)	)				3.41)
Monaco	0.03 (0.01-0.06)	0.02 (0-0.03)	-0.33	0.04 (0.01-0.08)	0.02 (0-0.03)	-3.3 (-3.64--2.95)
Mongolia	1.25 (0.24-2.68)	2.51 (0.54-5.57)	1.01	0.11 (0.02-0.22)	0.08 (0.02-0.18)	-1.34 (-1.61--1.08)
Montenegro	0.14 (0.03-0.29)	0.11 (0.03-0.22)	-0.21	0.02 (0-0.05)	0.01 (0-0.02)	-2.3 (-2.74--1.86)
Morocco	7.88 (1.63-23.67)	8.1 (1.9-18.92)	0.03	0.06 (0.01-0.18)	0.02 (0.01-0.06)	-2.31 (-2.67--1.96)
Mozambique	7.84 (1.88-15.42)	15.98 (3.53-30.04)	1.04	0.15 (0.04-0.28)	0.15 (0.03-0.28)	0.81 (0.52-1.1)
Myanmar	20.81 (5.33-36.62)	12.16 (2.91-21.62)	-0.42	0.09 (0.02-0.16)	0.03 (0.01-0.05)	-4.57 (-4.86--4.29)
Namibia	1.05 (0.23-1.99)	1.38 (0.3-2.79)	0.31	0.16 (0.04-0.3)	0.09 (0.02-0.19)	-2.04 (-2.59--1.49)
Nauru	0.01 (0-0.01)	0 (0-0.01)	-1	0.11 (0.02-0.2)	0.06 (0.01-0.12)	-2.12 (-2.3--1.93)
Nepal	50.09 (10.58-93.01)	42.12 (10.05-80.18)	-0.16	0.55 (0.12-1)	0.18 (0.04-0.35)	-3.78 (-3.87--3.69)
Netherlands	1.58 (0.37-2.8)	0.83 (0.2-1.47)	-0.47	0.01 (0-0.01)	0 (0-0)	-4.65 (-4.9--4.39)
New Zealand	0.45 (0.1-0.82)	0.12 (0.03-0.22)	-0.73	0.01 (0-0.02)	0 (0-0)	-6.32 (-7.16--5.47)
Nicaragua	0.4 (0.1-0.69)	0.35 (0.08-0.63)	-0.13	0.02 (0.01-0.04)	0.01 (0-0.01)	-4.42 (-4.56--4.29)
Niger	10.95 (2.35-20.79)	19.32 (4.48-39.58)	0.76	0.39 (0.08-0.73)	0.23 (0.05-0.46)	-1.91 (-2.09--1.73)
Nigeria	83.05 (19.26-147.43)	102.23 (24.66-184.57)	0.23	0.2 (0.05-0.35)	0.12 (0.03-0.21)	-1.99 (-2.36--1.62)
Niue	0 (0-0)	0 (0-0)	NA	0.02	0.02 (0-0.03)	-1.24

				(0.01-0.05)		(-1.37--1.11)
North Macedonia	0.73 (0.17-1.37)	0.28 (0.06-0.53)	-0.62	0.04 (0.01-0.07)	0.01 (0-0.02)	-4.33 (-4.67--4)
Northern Mariana Islands	0.02 (0-0.04)	0.01 (0-0.03)	-0.5	0.1 (0.02-0.18)	0.03 (0.01-0.05)	-4.29 (-4.63--3.96)
Norway	3.5 (0.74-6.48)	2.11 (0.48-3.83)	-0.4	0.05 (0.01-0.09)	0.02 (0-0.03)	-3.08 (-3.47--2.7)
Oman	0.04 (0.01-0.08)	0.04 (0.01-0.08)	0	0.01 (0-0.01)	0 (0-0)	-2.98 (-3.39--2.57)
Pakistan	353.88 (80.39-679.11)	454.86 (104.28-896.98)	0.29	0.63 (0.14-1.21)	0.33 (0.08-0.66)	-2.32 (-2.74--1.89)
Palau	0 (0-0.01)	0 (0-0.01)	NA	0.03 (0.01-0.06)	0.02 (0.01-0.04)	-1.26 (-1.4--1.12)
Palestine	0.04 (0.01-0.08)	0.03 (0.01-0.06)	-0.25	0.01 (0-0.01)	0 (0-0)	-4.08 (-4.34--3.82)
Panama	0.35 (0.08-0.61)	0.33 (0.08-0.59)	-0.06	0.02 (0.01-0.04)	0.01 (0-0.01)	-3.76 (-4.11--3.41)
Papua New Guinea	1.58 (0.39-3.06)	2.49 (0.52-4.53)	0.58	0.09 (0.02-0.17)	0.05 (0.01-0.09)	-1.97 (-2.02--1.92)
Paraguay	0.33 (0.07-0.59)	0.61 (0.15-1.16)	0.85	0.01 (0-0.03)	0.01 (0-0.02)	-0.99 (-1.1--0.89)
Peru	1.99 (0.46-3.42)	1.74 (0.4-3.24)	-0.13	0.02 (0-0.03)	0.01 (0-0.01)	-4.17 (-4.92--3.42)
Philippines	13.57 (3.33-24.26)	35.15 (8.42-61.45)	1.59	0.05 (0.01-0.08)	0.04 (0.01-0.07)	0.19 (-0.02-0.4)
Poland	10.3 (2.53-18.52)	4.08 (1.07-7.44)	-0.6	0.02 (0.01-0.04)	0.01 (0-0.01)	-4.15 (-4.48--3.83)
Portugal	3.07 (0.69-5.43)	1.69 (0.43-3.1)	-0.45	0.02 (0.01-0.04)	0.01 (0-0.01)	-4.71 (-5.04--4.38)

Puerto Rico	0.24 (0.06-0.42)	0.06 (0.02-0.11)	-0.75	0.01 (0-0.01)	0 (0-0)	-5.68 (-5.95--5.4)
Qatar	0.04 (0.01-0.07)	0.1 (0.02-0.18)	1.5	0.04 (0.01-0.07)	0.01 (0-0.03)	-3.83 (-4.33--3.33)
Republic of Korea	35.32 (7.75-63.51)	18.34 (4.23-33.06)	-0.48	0.13 (0.03-0.24)	0.02 (0-0.04)	-5.95 (-6.18--5.73)
Republic of Moldova	1.55 (0.32-3.04)	1.44 (0.34-2.66)	-0.07	0.03 (0.01-0.07)	0.03 (0.01-0.05)	-1 (-2.27-0.29)
Romania	8.06 (1.75-14.93)	13.94 (3.31-25.34)	0.73	0.03 (0.01-0.06)	0.04 (0.01-0.08)	0.95 (0.31-1.59)
Russian Federation	305.08 (52.6-620.75)	61.17 (13.43-114.45)	-0.8	0.17 (0.03-0.34)	0.03 (0.01-0.05)	-7.33 (-8.94--5.68)
Rwanda	4.54 (1.03-8.2)	2.88 (0.66-5.62)	-0.37	0.17 (0.04-0.31)	0.05 (0.01-0.1)	-5.06 (-5.51--4.61)
Saint Kitts and Nevis	0 (0-0.01)	0 (0-0)	NA	0.01 (0-0.02)	0 (0-0)	-4.84 (-5.4--4.27)
Saint Lucia	0.01 (0-0.02)	0.01 (0-0.01)	0	0.01 (0-0.02)	0 (0-0.01)	-4.72 (-5.11--4.33)
Saint Vincent and the Grenadines	0.01 (0-0.01)	0 (0-0.01)	-1	0.01 (0-0.01)	0 (0-0)	-3.54 (-3.89--3.18)
Samoa	0.03 (0.01-0.06)	0.04 (0.01-0.07)	0.33	0.04 (0.01-0.07)	0.03 (0.01-0.05)	-0.6 (-0.94--0.25)
San Marino	0 (0-0.01)	0 (0-0)	NA	0.01 (0-0.02)	0 (0-0.01)	-3.81 (-4.06--3.56)
Sao Tome and Principe	0.05 (0.01-0.1)	0.07 (0.02-0.13)	0.4	0.09 (0.02-0.16)	0.06 (0.02-0.12)	-1.46 (-1.74--1.17)
Saudi Arabia	2.1 (0.43-3.81)	3.83 (0.94-7.38)	0.82	0.03 (0.01-0.06)	0.01 (0-0.03)	-3.38 (-3.59--3.16)
Senegal	10.18 (2.26-17.9	12.27 (2.95-22.8	0.21	0.33 (0.07-0.57)	0.16 (0.04-0.3)	-2.23 (-2.38-

	5)	6)				-2.09)
Serbia	2.15 (0.47-4.13)	0.71 (0.18-1.34)	-0.67	0.02 (0-0.04)	0 (0-0.01)	-5.44 (-5.81--5.06)
Seychelles	0.01 (0-0.01)	0.01 (0-0.01)	0	0.01 (0-0.02)	0.01 (0-0.01)	-3.83 (-4.12--3.53)
Sierra Leone	3.57 (0.83-6.37)	4.83 (1.06-9.49)	0.35	0.18 (0.04-0.33)	0.13 (0.03-0.25)	-0.77 (-1.14--0.39)
Singapore	0.64 (0.16-1.14)	0.36 (0.09-0.63)	-0.44	0.03 (0.01-0.06)	0 (0-0.01)	-6.11 (-6.32--5.91)
Slovakia	0.91 (0.23-1.65)	0.48 (0.11-0.88)	-0.47	0.02 (0-0.03)	0.01 (0-0.01)	-3.22 (-3.32--3.11)
Slovenia	0.88 (0.18-1.68)	0.26 (0.06-0.45)	-0.7	0.04 (0.01-0.07)	0.01 (0-0.01)	-5.5 (-6.3--4.7)
Solomon Islands	0.04 (0.01-0.08)	0.06 (0.02-0.12)	0.5	0.03 (0.01-0.06)	0.02 (0-0.03)	-2.18 (-2.33--2.03)
Somalia	6.35 (1.38-13.1)	15.72 (3.86-31)	1.48	0.28 (0.06-0.54)	0.29 (0.07-0.57)	0.06 (-0.05-0.17)
South Africa	15.05 (3.34-27.58)	36.94 (9.07-63.33)	1.45	0.07 (0.01-0.12)	0.07 (0.02-0.13)	0.86 (-0.01-1.75)
South Sudan	4.08 (0.97-8.09)	6.22 (1.48-12.03)	0.52	0.17 (0.04-0.33)	0.17 (0.04-0.33)	-0.24 (-0.44--0.05)
Spain	18.26 (3.93-33.69)	4.27 (0.99-8.13)	-0.77	0.03 (0.01-0.06)	0 (0-0.01)	-6.96 (-7.36--6.56)
Sri Lanka	1.04 (0.24-1.81)	0.8 (0.18-1.53)	-0.23	0.01 (0-0.02)	0 (0-0.01)	-3.85 (-4.19--3.52)
Sudan	1.95 (0.38-3.71)	1.46 (0.34-2.87)	-0.25	0.02 (0-0.04)	0.01 (0-0.01)	-3.49 (-3.61--3.38)
Suriname	0.03 (0.01-0.05)	0.03 (0.01-0.05)	0	0.01 (0-0.02)	0 (0-0.01)	-2.89 (-3.24--2.53)
Sweden	2.87	1.22	-0.57	0.02 (0-0.03)	0 (0-0.01)	-4.4

	(0.61-5.1)	(0.27-2.31)				(-4.82--3.97)
Switzerland	0.97 (0.21-1.71)	0.31 (0.08-0.55)	-0.68	0.01 (0-0.02)	0 (0-0)	-5.72 (-5.91--5.53)
Syrian Arab Republic	0.25 (0.06-0.45)	0.2 (0.05-0.38)	-0.2	0.01 (0-0.01)	0 (0-0)	-4.09 (-4.38--3.8)
Taiwan	6.97 (1.67-12.54)	2.52 (0.64-4.28)	-0.64	0.05 (0.01-0.09)	0.01 (0-0.01)	-7.47 (-7.79--7.15)
Tajikistan	1.93 (0.43-3.58)	2.67 (0.6-4.82)	0.38	0.06 (0.01-0.12)	0.04 (0.01-0.07)	-2.22 (-2.61--1.83)
Thailand	8.27 (2.09-14.67)	7.73 (1.77-14.3)	-0.07	0.03 (0.01-0.05)	0.01 (0-0.01)	-4.44 (-4.57--4.3)
Timor-Leste	0.14 (0.03-0.26)	0.27 (0.06-0.52)	0.93	0.06 (0.01-0.11)	0.03 (0.01-0.06)	-1.98 (-2.13--1.83)
Togo	1.9 (0.43-3.52)	4.61 (1.09-9.01)	1.43	0.17 (0.04-0.32)	0.14 (0.03-0.26)	-0.75 (-1.06--0.45)
Tokelau	0 (0-0)	0 (0-0)	NA	0.04 (0.01-0.09)	0.02 (0-0.03)	-3.42 (-3.61--3.22)
Tonga	0.01 (0-0.02)	0.01 (0-0.02)	0	0.02 (0-0.04)	0.01 (0-0.02)	-1.41 (-1.56--1.26)
Trinidad and Tobago	0.05 (0.01-0.09)	0.04 (0.01-0.07)	-0.2	0.01 (0-0.01)	0 (0-0)	-3.68 (-4.05--3.32)
Tunisia	0.37 (0.08-0.69)	0.4 (0.09-0.81)	0.08	0.01 (0-0.01)	0 (0-0.01)	-3.04 (-3.19--2.89)
T 眉 rkiye	4.3 (0.91-8.23)	2.23 (0.55-4.12)	-0.48	0.01 (0-0.03)	0 (0-0)	-5.87 (-6.42--5.31)
Turkmenistan	0.82 (0.19-1.5)	2.24 (0.52-4.22)	1.73	0.04 (0.01-0.07)	0.05 (0.01-0.09)	-0.2 (-0.9-0.51)
Tuvalu	0.01 (0-0.02)	0 (0-0.01)	-1	0.12 (0.03-0.22)	0.04 (0.01-0.07)	-3.31 (-3.41--3.2)

Uganda	7.45 (1.62-15.31)	11.98 (2.73-23.71)	0.61	0.12 (0.03-0.25)	0.08 (0.02-0.16)	-1.94 (-2.4--1.47)
Ukraine	7.63 (1.69-14.15)	9.82 (2.23-18.15)	0.29	0.01 (0-0.02)	0.01 (0-0.03)	1.18 (0.19-2.17)
United Arab Emirates	0.09 (0.02-0.2)	0.18 (0.03-0.37)	1	0.02 (0-0.03)	0.01 (0-0.02)	-2.09 (-2.33--1.85)
United Kingdom	7.99 (1.81-14.49)	4.22 (1.01-7.62)	-0.47	0.01 (0-0.02)	0 (0-0.01)	-2.96 (-3.22--2.7)
United Republic of Tanzania	11.53 (2.5-21.71)	17.67 (3.91-32.74)	0.53	0.12 (0.03-0.22)	0.07 (0.02-0.13)	-1.73 (-1.86--1.6)
United States of America	58.49 (13.78-107.54)	32.07 (7.76-56.89)	-0.45	0.02 (0-0.03)	0.01 (0-0.01)	-4.22 (-4.62--3.82)
United States Virgin Islands	0 (0-0.01)	0 (0-0.01)	NA	0 (0-0.01)	0 (0-0)	-1.82 (-2.06--1.59)
Uruguay	0.66 (0.15-1.21)	0.49 (0.11-0.91)	-0.26	0.02 (0-0.03)	0.01 (0-0.02)	-1.71 (-2.08--1.33)
Uzbekistan	3.32 (0.71-5.89)	5.97 (1.32-10.61)	0.8	0.03 (0.01-0.05)	0.02 (0-0.03)	-1.35 (-2.17--0.53)
Vanuatu	0.03 (0.01-0.08)	0.06 (0.01-0.12)	1	0.05 (0.01-0.12)	0.03 (0.01-0.06)	-1.56 (-1.65--1.47)
Venezuela (Bolivarian Republic of)	1.79 (0.42-3.02)	2.24 (0.51-4.09)	0.25	0.02 (0-0.03)	0.01 (0-0.01)	-3.29 (-3.88--2.69)
Viet Nam	9.64 (2.34-17.68)	7.9 (1.8-14.15)	-0.18	0.03 (0.01-0.05)	0.01 (0-0.02)	-3.41 (-3.49--3.33)
Yemen	0.64 (0.12-1.32)	0.8 (0.16-1.6)	0.25	0.01 (0-0.03)	0.01 (0-0.01)	-2.99 (-3.22--2.77)
Zambia	7.01 (1.59-12.33)	4.41 (0.94-8.69)	-0.37	0.25 (0.06-0.44)	0.07 (0.02-0.13)	-5.37 (-6.18--4.55)
Zimbabwe	3.47 (0.76-6.09	10.06 (2.45-18.6	1.9	0.09 (0.02-0.15)	0.14 (0.03-0.25)	2.5 (1.78-3

)) .24)

Supplementary table 2. The deaths of TB attributable to DHPM cases and rates in 1990 and 2021 across 204 countries, and the trends from 1990 to 2021.
