# Supplementary material for: The global burden of tuberculosis attributable to diet high in processed meat from 1990 to 2021: findings from the Global Burden of Disease Study 2021
Source: Front Nutr. 2026 Jan 20;12:1666550. doi: 10.3389/fnut.2025.1666550 (PMC12864051; doi:10.3389/fnut.2025.1666550)
Supplement: Supplementary file 2 [file Image_1.pdf]

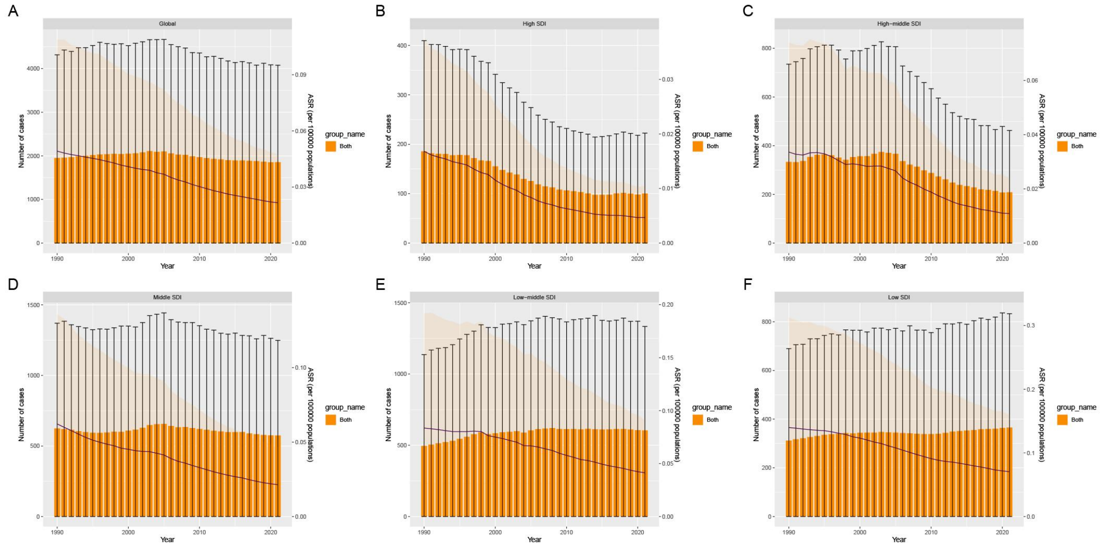

Supplementary figure 1. Death cases and ASMR of TB attributable to DHPM from 1990 to 2021.

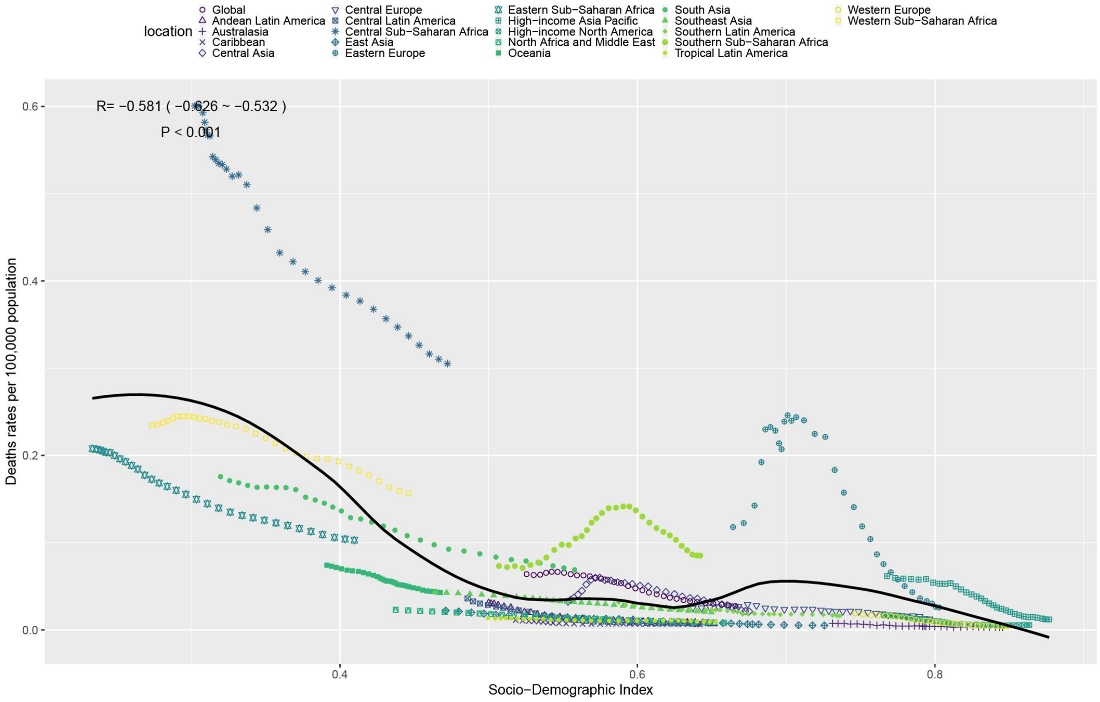

Supplementary figure 2. ASMR of TB attributable to DHPM in 21 GBD regions by SDI, 1990–2021.
